# Supplementary material for: Genetically predicted high IGF-1 levels showed protective effects on COVID-19 susceptibility and hospitalization: a Mendelian randomisation study with data from 60 studies across 25 countries
Source: eLife. 2022 Oct 17;11:e79720. doi: 10.7554/eLife.79720 (PMC9576268; doi:10.7554/eLife.79720)
Supplement: Supplementary file 1. [file elife-79720-supp1.docx]

**Supplementary Online Content**

supplementary file 1a. Genetic instruments for sex hormone and IGF-1

supplementary file 1b. Genetic instruments for estradiol in male and female

supplementary file 1c. Genetic instruments for testosterone in male

supplementary file 1d. Genetic instruments for testosterone in female

**supplementary file 1a. Genetic instruments for sex hormone and IGF-1**

| **Phenotype** | **SNP** | **Chr** | **Position** | **REF** | **ALT** | **MAF** | **Beta** | **SE** | ***p*-value** |
| --- | --- | --- | --- | --- | --- | --- | --- | --- | --- |
| Testosterone | rs182050989 | 1 | 27262545 | C | T | 0.971 | 0.027 | 0.004 | 1.20E-12 |
| Testosterone | rs4453027 | 1 | 41453453 | G | T | 0.575 | 0.010 | 0.001 | 5.50E-15 |
| Testosterone | rs4912377 | 1 | 57015121 | C | A | 0.487 | 0.008 | 0.001 | 1.40E-09 |
| Testosterone | rs12745935 | 1 | 59644082 | G | A | 0.300 | 0.008 | 0.001 | 1.50E-08 |
| Testosterone | rs6684361 | 1 | 101737743 | C | T | 0.308 | 0.025 | 0.001 | 2.50E-70 |
| Testosterone | rs1977658 | 1 | 107607037 | T | G | 0.344 | 0.015 | 0.001 | 1.60E-27 |
| Testosterone | rs41264630 | 1 | 149885800 | A | G | 0.080 | 0.014 | 0.002 | 3.90E-09 |
| Testosterone | rs267733 | 1 | 150958836 | A | G | 0.839 | 0.012 | 0.002 | 1.20E-12 |
| Testosterone | rs1870940 | 1 | 154984363 | G | A | 0.728 | 0.010 | 0.001 | 2.90E-12 |
| Testosterone | rs72708239 | 1 | 155985899 | A | G | 0.739 | 0.010 | 0.001 | 1.50E-10 |
| Testosterone | rs733190 | 1 | 172095226 | T | C | 0.526 | 0.008 | 0.001 | 1.60E-09 |
| Testosterone | rs61320678 | 1 | 214177319 | G | T | 0.236 | 0.010 | 0.002 | 2.30E-11 |
| Testosterone | rs78851238 | 1 | 216876964 | C | T | 0.172 | 0.009 | 0.002 | 5.00E-08 |
| Testosterone | rs61823391 | 1 | 218541992 | T | C | 0.683 | 0.011 | 0.001 | 4.20E-16 |
| Testosterone | rs62115715 | 2 | 12531863 | C | T | 0.028 | 0.030 | 0.004 | 8.30E-14 |
| Testosterone | rs3771243 | 2 | 20412227 | A | G | 0.389 | 0.009 | 0.001 | 2.50E-12 |
| Testosterone | rs1260326 | 2 | 27730940 | C | T | 0.606 | 0.028 | 0.001 | 3.50E-103 |
| Testosterone | rs2374456 | 2 | 43271621 | G | C | 0.585 | 0.008 | 0.001 | 1.00E-09 |
| Testosterone | rs58839393 | 2 | 43490619 | A | T | 0.839 | 0.016 | 0.002 | 3.90E-19 |
| Testosterone | rs111861797 | 2 | 48022688 | T | C | 0.807 | 0.012 | 0.002 | 3.30E-13 |
| Testosterone | rs11888201 | 2 | 60066405 | C | G | 0.542 | 0.007 | 0.001 | 3.30E-08 |
| Testosterone | rs2723065 | 2 | 65279414 | G | A | 0.375 | 0.011 | 0.001 | 1.30E-14 |
| Testosterone | rs10202148 | 2 | 86163767 | G | A | 0.739 | 0.009 | 0.001 | 2.70E-10 |
| Testosterone | rs590097 | 2 | 111934107 | G | T | 0.647 | 0.019 | 0.001 | 3.80E-48 |
| Testosterone | rs62162863 | 2 | 112071459 | G | T | 0.577 | 0.010 | 0.001 | 6.60E-11 |
| Testosterone | rs1128249 | 2 | 165528624 | T | G | 0.393 | 0.009 | 0.001 | 4.10E-13 |
| Testosterone | rs56117787 | 2 | 178154343 | G | A | 0.199 | 0.013 | 0.002 | 1.90E-14 |
| Testosterone | rs2011425 | 2 | 234627608 | T | G | 0.920 | 0.025 | 0.002 | 3.10E-26 |
| Testosterone | rs7618363 | 3 | 10545125 | C | G | 0.841 | 0.012 | 0.002 | 1.30E-10 |
| Testosterone | rs3103310 | 3 | 12473045 | A | G | 0.758 | 0.009 | 0.002 | 2.70E-09 |
| Testosterone | rs73075656 | 3 | 41105947 | G | T | 0.867 | 0.015 | 0.002 | 2.10E-15 |
| Testosterone | rs57615517 | 3 | 52322417 | G | A | 0.685 | 0.008 | 0.001 | 4.70E-10 |
| Testosterone | rs696516 | 3 | 136067778 | G | T | 0.266 | 0.008 | 0.001 | 1.10E-09 |
| Testosterone | rs4678408 | 3 | 138053187 | G | A | 0.629 | 0.009 | 0.001 | 5.90E-12 |
| Testosterone | rs9850919 | 3 | 169177924 | C | T | 0.406 | 0.007 | 0.001 | 4.70E-08 |
| Testosterone | rs56271032 | 3 | 172154210 | G | A | 0.131 | 0.011 | 0.002 | 3.60E-09 |
| Testosterone | rs36205397 | 4 | 3470604 | G | A | 0.421 | 0.010 | 0.001 | 1.30E-13 |
| Testosterone | rs7686914 | 4 | 69537915 | C | T | 0.475 | 0.011 | 0.001 | 9.20E-18 |
| Testosterone | rs4632729 | 4 | 69946004 | A | G | 0.545 | 0.015 | 0.001 | 2.10E-30 |
| Testosterone | rs13152154 | 4 | 77417756 | C | T | 0.273 | 0.008 | 0.001 | 7.30E-09 |
| Testosterone | rs1408 | 4 | 88057353 | G | A | 0.422 | 0.011 | 0.001 | 6.80E-19 |
| Testosterone | rs10017280 | 4 | 104609388 | C | T | 0.870 | 0.012 | 0.002 | 1.20E-09 |
| Testosterone | rs2903385 | 4 | 106094427 | A | G | 0.485 | 0.009 | 0.001 | 2.10E-14 |
| Testosterone | rs4245930 | 4 | 109038654 | G | A | 0.367 | 0.007 | 0.001 | 2.40E-08 |
| Testosterone | rs62314881 | 4 | 113956403 | G | T | 0.817 | 0.011 | 0.002 | 2.10E-10 |
| Testosterone | rs11937496 | 4 | 149613443 | G | A | 0.556 | 0.007 | 0.001 | 2.90E-08 |
| Testosterone | rs112694713 | 5 | 35247932 | A | G | 0.987 | 0.041 | 0.006 | 3.50E-13 |
| Testosterone | rs9686661 | 5 | 55861786 | C | T | 0.800 | 0.012 | 0.002 | 1.30E-14 |
| Testosterone | rs4431325 | 5 | 76461706 | T | C | 0.059 | 0.021 | 0.003 | 9.90E-15 |
| Testosterone | rs77741622 | 5 | 76491857 | G | A | 0.653 | 0.009 | 0.001 | 9.20E-11 |
| Testosterone | rs784420 | 5 | 77987524 | G | A | 0.288 | 0.013 | 0.001 | 3.20E-21 |
| Testosterone | rs112530420 | 5 | 95871370 | C | T | 0.166 | 0.010 | 0.002 | 2.30E-08 |
| Testosterone | rs12658172 | 5 | 124205385 | G | C | 0.842 | 0.018 | 0.002 | 2.70E-23 |
| Testosterone | rs4835948 | 5 | 127870190 | T | C | 0.533 | 0.007 | 0.001 | 2.90E-09 |
| Testosterone | rs13185520 | 5 | 137807260 | A | G | 0.575 | 0.008 | 0.001 | 9.10E-10 |
| Testosterone | rs62394296 | 6 | 25870381 | T | C | 0.117 | 0.012 | 0.002 | 4.20E-10 |
| Testosterone | rs9272309 | 6 | 32603936 | A | G | 0.620 | 0.013 | 0.001 | 1.10E-19 |
| Testosterone | rs6939861 | 6 | 41703041 | G | A | 0.738 | 0.009 | 0.001 | 3.40E-09 |
| Testosterone | rs6458331 | 6 | 43280028 | T | C | 0.725 | 0.014 | 0.001 | 1.20E-22 |
| Testosterone | rs57323441 | 6 | 52404343 | C | A | 0.123 | 0.011 | 0.002 | 2.20E-09 |
| Testosterone | rs1681967 | 6 | 64270790 | A | G | 0.918 | 0.015 | 0.002 | 5.90E-10 |
| Testosterone | rs221584 | 6 | 99739942 | G | A | 0.817 | 0.010 | 0.002 | 1.60E-08 |
| Testosterone | rs7759938 | 6 | 105378954 | T | C | 0.678 | 0.010 | 0.001 | 1.70E-13 |
| Testosterone | rs36182456 | 6 | 119167949 | A | G | 0.779 | 0.018 | 0.002 | 2.10E-33 |
| Testosterone | rs577721086 | 6 | 127440047 | T | C | 0.950 | 0.020 | 0.003 | 3.40E-11 |
| Testosterone | rs520829 | 6 | 160767905 | G | T | 0.475 | 0.010 | 0.001 | 2.20E-16 |
| Testosterone | rs12702516 | 7 | 6526006 | A | C | 0.152 | 0.011 | 0.002 | 3.70E-09 |
| Testosterone | rs10278686 | 7 | 15031450 | T | C | 0.491 | 0.010 | 0.001 | 1.30E-14 |
| Testosterone | rs6462989 | 7 | 40869754 | A | C | 0.659 | 0.008 | 0.001 | 1.50E-09 |
| Testosterone | rs34060476 | 7 | 73037956 | G | A | 0.134 | 0.020 | 0.002 | 5.30E-26 |
| Testosterone | rs445 | 7 | 92408370 | C | T | 0.905 | 0.012 | 0.002 | 1.20E-08 |
| Testosterone | rs45446698 | 7 | 99332948 | T | G | 0.958 | 0.120 | 0.003 | 1.00E-200 |
| Testosterone | rs11556924 | 7 | 129663496 | T | C | 0.389 | 0.008 | 0.001 | 1.20E-09 |
| Testosterone | rs157934 | 7 | 130585492 | C | T | 0.305 | 0.008 | 0.001 | 3.40E-09 |
| Testosterone | rs2306847 | 7 | 137798593 | T | A | 0.791 | 0.019 | 0.002 | 5.20E-34 |
| Testosterone | rs4725944 | 7 | 150476673 | C | G | 0.390 | 0.007 | 0.001 | 4.60E-08 |
| Testosterone | rs9638084 | 7 | 156311745 | G | A | 0.604 | 0.007 | 0.001 | 1.60E-08 |
| Testosterone | rs73200740 | 8 | 10627311 | A | C | 0.236 | 0.009 | 0.002 | 1.80E-09 |
| Testosterone | rs201643157 | 8 | 23399890 | T | C | 0.250 | 0.009 | 0.001 | 9.00E-10 |
| Testosterone | rs881301 | 8 | 38332318 | T | C | 0.587 | 0.008 | 0.001 | 6.40E-10 |
| Testosterone | rs35222808 | 8 | 77879704 | C | A | 0.748 | 0.009 | 0.001 | 5.70E-10 |
| Testosterone | rs150539196 | 8 | 81399180 | G | A | 0.036 | 0.022 | 0.004 | 3.50E-10 |
| Testosterone | rs34955534 | 8 | 81710349 | G | A | 0.901 | 0.015 | 0.002 | 6.50E-12 |
| Testosterone | rs35783704 | 8 | 105966258 | A | G | 0.101 | 0.020 | 0.002 | 2.60E-20 |
| Testosterone | rs6471583 | 8 | 143991092 | G | A | 0.443 | 0.011 | 0.001 | 1.10E-19 |
| Testosterone | rs12683780 | 9 | 16252807 | A | C | 0.669 | 0.010 | 0.001 | 2.80E-13 |
| Testosterone | rs4961485 | 9 | 16360889 | T | C | 0.933 | 0.016 | 0.003 | 5.90E-10 |
| Testosterone | rs17810415 | 9 | 19032907 | G | A | 0.200 | 0.010 | 0.002 | 6.70E-09 |
| Testosterone | rs10971921 | 9 | 34111002 | A | G | 0.131 | 0.014 | 0.002 | 7.40E-13 |
| Testosterone | rs1547308 | 9 | 114603240 | C | T | 0.800 | 0.012 | 0.002 | 6.80E-15 |
| Testosterone | rs10817260 | 9 | 114828332 | C | T | 0.810 | 0.014 | 0.002 | 6.90E-17 |
| Testosterone | rs10982192 | 9 | 117149417 | T | C | 0.221 | 0.009 | 0.002 | 2.20E-08 |
| Testosterone | rs494242 | 9 | 136145118 | C | T | 0.660 | 0.008 | 0.001 | 3.10E-09 |
| Testosterone | rs35182096 | 9 | 137268682 | C | T | 0.256 | 0.008 | 0.001 | 3.60E-08 |
| Testosterone | rs36032941 | 10 | 5062752 | C | A | 0.704 | 0.023 | 0.001 | 5.00E-56 |
| Testosterone | rs1171617 | 10 | 61467182 | T | G | 0.767 | 0.017 | 0.002 | 8.50E-31 |
| Testosterone | rs2675611 | 10 | 63644246 | T | C | 0.469 | 0.007 | 0.001 | 3.40E-08 |
| Testosterone | rs10740131 | 10 | 65271488 | T | A | 0.473 | 0.038 | 0.001 | 2.50E-196 |
| Testosterone | rs1204083 | 10 | 69835197 | C | G | 0.346 | 0.012 | 0.001 | 2.80E-18 |
| Testosterone | rs1782652 | 10 | 81074125 | T | A | 0.619 | 0.008 | 0.001 | 9.50E-09 |
| Testosterone | rs11572082 | 10 | 96826922 | C | G | 0.878 | 0.013 | 0.002 | 8.00E-11 |
| Testosterone | rs11191801 | 10 | 105532165 | A | C | 0.707 | 0.009 | 0.001 | 6.00E-12 |
| Testosterone | rs74440003 | 10 | 111995456 | A | T | 0.261 | 0.008 | 0.001 | 1.10E-08 |
| Testosterone | rs11564722 | 11 | 2178330 | T | C | 0.240 | 0.009 | 0.002 | 8.20E-09 |
| Testosterone | rs7937758 | 11 | 10236478 | A | G | 0.504 | 0.007 | 0.001 | 2.90E-08 |
| Testosterone | rs11023881 | 11 | 16246700 | T | A | 0.612 | 0.009 | 0.001 | 1.70E-11 |
| Testosterone | rs2035838 | 11 | 29200384 | A | G | 0.852 | 0.011 | 0.002 | 2.50E-10 |
| Testosterone | rs12294104 | 11 | 30382899 | T | C | 0.173 | 0.009 | 0.002 | 9.40E-09 |
| Testosterone | rs171021 | 11 | 72317557 | C | T | 0.703 | 0.014 | 0.001 | 2.70E-21 |
| Testosterone | rs12796488 | 11 | 94131557 | C | A | 0.824 | 0.010 | 0.002 | 1.40E-08 |
| Testosterone | rs4938576 | 11 | 118746769 | G | T | 0.588 | 0.007 | 0.001 | 1.50E-08 |
| Testosterone | rs10892924 | 11 | 122773715 | T | A | 0.568 | 0.011 | 0.001 | 2.70E-17 |
| Testosterone | rs12788072 | 11 | 123334298 | G | A | 0.178 | 0.010 | 0.002 | 2.70E-08 |
| Testosterone | rs618888 | 11 | 125081521 | T | G | 0.283 | 0.008 | 0.001 | 3.50E-08 |
| Testosterone | rs56196860 | 12 | 2908330 | A | C | 0.031 | 0.064 | 0.004 | 7.20E-71 |
| Testosterone | rs180435 | 12 | 47180370 | G | C | 0.809 | 0.010 | 0.002 | 3.00E-09 |
| Testosterone | rs2583949 | 12 | 66194243 | C | T | 0.895 | 0.012 | 0.002 | 5.00E-09 |
| Testosterone | rs9509847 | 13 | 22318314 | C | A | 0.628 | 0.020 | 0.001 | 1.60E-52 |
| Testosterone | rs41284816 | 13 | 50655989 | T | G | 0.019 | 0.026 | 0.005 | 1.50E-08 |
| Testosterone | rs9543012 | 13 | 73172648 | C | T | 0.353 | 0.008 | 0.001 | 2.60E-10 |
| Testosterone | rs2038695 | 13 | 100559123 | C | A | 0.449 | 0.007 | 0.001 | 2.10E-08 |
| Testosterone | rs72660136 | 13 | 109960307 | T | C | 0.966 | 0.021 | 0.004 | 8.50E-10 |
| Testosterone | rs7342537 | 14 | 21555063 | G | A | 0.018 | 0.036 | 0.005 | 5.50E-13 |
| Testosterone | rs2256191 | 14 | 64656855 | T | C | 0.655 | 0.008 | 0.001 | 7.40E-09 |
| Testosterone | rs28929474 | 14 | 94844947 | T | C | 0.020 | 0.040 | 0.005 | 1.10E-17 |
| Testosterone | rs12436785 | 14 | 98550490 | C | T | 0.416 | 0.011 | 0.001 | 7.00E-16 |
| Testosterone | rs28576256 | 14 | 99718173 | G | A | 0.869 | 0.013 | 0.002 | 9.10E-12 |
| Testosterone | rs11629457 | 14 | 100805583 | T | C | 0.265 | 0.008 | 0.001 | 1.20E-08 |
| Testosterone | rs73365510 | 14 | 106875035 | A | G | 0.071 | 0.022 | 0.003 | 4.00E-12 |
| Testosterone | rs7183977 | 15 | 40377092 | C | T | 0.347 | 0.021 | 0.001 | 3.10E-57 |
| Testosterone | rs55707100 | 15 | 43820717 | C | T | 0.975 | 0.027 | 0.004 | 7.70E-12 |
| Testosterone | rs79391862 | 15 | 53739426 | A | C | 0.986 | 0.041 | 0.006 | 2.40E-14 |
| Testosterone | rs35698268 | 15 | 60978246 | G | C | 0.224 | 0.010 | 0.002 | 4.60E-10 |
| Testosterone | rs12914034 | 15 | 65941010 | A | T | 0.637 | 0.007 | 0.001 | 4.70E-08 |
| Testosterone | rs2201003 | 15 | 75463144 | A | G | 0.364 | 0.008 | 0.001 | 6.70E-10 |
| Testosterone | rs4464040 | 15 | 79840557 | C | T | 0.850 | 0.014 | 0.002 | 2.80E-16 |
| Testosterone | rs35816571 | 15 | 85554122 | C | G | 0.829 | 0.014 | 0.002 | 1.40E-16 |
| Testosterone | rs1822246 | 15 | 96245020 | A | G | 0.703 | 0.008 | 0.001 | 1.60E-09 |
| Testosterone | rs56332871 | 15 | 96714816 | A | C | 0.273 | 0.017 | 0.001 | 3.50E-34 |
| Testosterone | rs370222 | 16 | 4157696 | G | A | 0.697 | 0.010 | 0.001 | 3.10E-12 |
| Testosterone | rs28495625 | 16 | 11891361 | A | C | 0.149 | 0.013 | 0.002 | 1.20E-12 |
| Testosterone | rs2764772 | 16 | 20060653 | A | T | 0.334 | 0.008 | 0.001 | 9.90E-10 |
| Testosterone | rs2061679 | 16 | 81573976 | T | C | 0.066 | 0.033 | 0.003 | 1.50E-36 |
| Testosterone | rs2287322 | 17 | 1641035 | G | A | 0.222 | 0.009 | 0.002 | 1.10E-08 |
| Testosterone | rs1799941 | 17 | 7533423 | A | G | 0.261 | 0.064 | 0.001 | 1.00E-200 |
| Testosterone | rs941446 | 17 | 17680273 | T | C | 0.632 | 0.008 | 0.001 | 3.50E-10 |
| Testosterone | rs55885610 | 17 | 27579011 | C | T | 0.964 | 0.023 | 0.004 | 2.60E-10 |
| Testosterone | rs9913470 | 17 | 29566575 | A | G | 0.803 | 0.014 | 0.002 | 5.30E-15 |
| Testosterone | rs1317701 | 17 | 47444794 | C | T | 0.312 | 0.017 | 0.001 | 3.70E-34 |
| Testosterone | rs28421540 | 18 | 3818842 | A | C | 0.715 | 0.011 | 0.001 | 3.00E-14 |
| Testosterone | rs17089026 | 18 | 71936165 | G | T | 0.023 | 0.053 | 0.004 | 7.10E-37 |
| Testosterone | rs79384925 | 18 | 71938618 | C | A | 0.863 | 0.026 | 0.002 | 2.00E-41 |
| Testosterone | rs759068 | 19 | 2792566 | A | G | 0.294 | 0.011 | 0.001 | 5.20E-16 |
| Testosterone | rs77017252 | 19 | 7218922 | T | A | 0.756 | 0.010 | 0.002 | 4.80E-12 |
| Testosterone | rs8111359 | 19 | 10471462 | C | T | 0.905 | 0.020 | 0.002 | 1.90E-19 |
| Testosterone | rs4804669 | 19 | 12502457 | A | G | 0.217 | 0.013 | 0.002 | 2.40E-17 |
| Testosterone | rs146497684 | 19 | 17346441 | T | A | 0.030 | 0.044 | 0.004 | 1.20E-29 |
| Testosterone | rs34858588 | 19 | 19457235 | C | G | 0.921 | 0.016 | 0.002 | 4.30E-10 |
| Testosterone | rs11673591 | 19 | 41985931 | A | T | 0.252 | 0.012 | 0.001 | 4.80E-17 |
| Testosterone | rs67967246 | 19 | 46408018 | C | A | 0.132 | 0.015 | 0.002 | 9.20E-16 |
| Testosterone | rs2241388 | 19 | 47572987 | T | C | 0.279 | 0.011 | 0.001 | 3.20E-14 |
| Testosterone | rs78248023 | 19 | 49515171 | A | C | 0.078 | 0.019 | 0.002 | 1.60E-16 |
| Testosterone | rs6130613 | 20 | 43054441 | T | C | 0.470 | 0.011 | 0.001 | 2.90E-15 |
| Testosterone | rs6020423 | 20 | 48909667 | C | T | 0.760 | 0.010 | 0.002 | 6.40E-12 |
| Testosterone | rs2273991 | 20 | 60588049 | G | C | 0.897 | 0.013 | 0.002 | 3.00E-09 |
| Testosterone | rs8126001 | 20 | 62711459 | T | C | 0.490 | 0.010 | 0.001 | 2.30E-14 |
| Testosterone | rs12185851 | 21 | 43372219 | C | T | 0.232 | 0.009 | 0.002 | 6.70E-09 |
| Testosterone | rs5752773 | 22 | 29105415 | G | C | 0.327 | 0.011 | 0.001 | 2.20E-14 |
| Testosterone | rs5763800 | 22 | 30533409 | A | G | 0.977 | 0.027 | 0.004 | 9.00E-10 |
| Testosterone | rs5751229 | 22 | 42545221 | A | G | 0.228 | 0.011 | 0.002 | 1.50E-12 |
| Testosterone | rs738409 | 22 | 44324727 | G | C | 0.216 | 0.015 | 0.002 | 7.40E-21 |
| Testosterone | rs41378347 | 22 | 46636976 | G | A | 0.888 | 0.015 | 0.002 | 5.20E-14 |
| Testosterone | rs62220604 | 22 | 49677464 | A | G | 0.283 | 0.008 | 0.001 | 1.60E-09 |
| Testosterone | rs10225221 | 7 | 35401635 | C | A | 0.299 | 0.005 | 0.001 | 3.10E-08 |
| Testosterone | rs1799831 | 7 | 44199142 | C | T | 0.844 | 0.009 | 0.001 | 1.60E-13 |
| Testosterone | rs7794048 | 7 | 46275882 | G | A | 0.937 | 0.013 | 0.002 | 4.80E-14 |
| Testosterone | rs6975610 | 7 | 46642734 | G | A | 0.448 | 0.006 | 0.001 | 4.50E-11 |
| Testosterone | rs12536766 | 7 | 70158864 | T | G | 0.570 | 0.006 | 0.001 | 4.10E-13 |
| Testosterone | rs11770446 | 7 | 77358072 | G | A | 0.288 | 0.007 | 0.001 | 1.40E-19 |
| Testosterone | rs1229492 | 7 | 81564122 | T | C | 0.268 | 0.011 | 0.001 | 1.30E-32 |
| Testosterone | rs445 | 7 | 92408370 | C | T | 0.905 | 0.012 | 0.001 | 4.80E-18 |
| Testosterone | rs7015 | 7 | 97920623 | G | A | 0.815 | 0.034 | 0.001 | 1.00E-200 |
| Testosterone | rs1859690 | 7 | 99227172 | G | A | 0.067 | 0.013 | 0.002 | 7.10E-16 |
| Testosterone | rs12667888 | 7 | 100474289 | C | T | 0.183 | 0.017 | 0.001 | 2.10E-59 |
| Testosterone | rs149092986 | 7 | 111624089 | T | C | 0.976 | 0.016 | 0.003 | 2.40E-08 |
| Testosterone | rs42374 | 7 | 116444070 | T | C | 0.547 | 0.006 | 0.001 | 8.60E-15 |
| Testosterone | rs34748838 | 7 | 130459242 | T | C | 0.490 | 0.008 | 0.001 | 2.30E-20 |
| Testosterone | rs157935 | 7 | 130585553 | G | T | 0.303 | 0.012 | 0.001 | 1.40E-39 |
| Testosterone | rs200087953 | 7 | 133575017 | A | G | 0.682 | 0.005 | 0.001 | 2.60E-08 |
| Testosterone | rs1559535 | 7 | 135143971 | G | A | 0.403 | 0.007 | 0.001 | 2.00E-17 |
| Testosterone | rs34372369 | 7 | 143092269 | A | G | 0.052 | 0.016 | 0.002 | 2.00E-15 |
| Testosterone | rs4725944 | 7 | 150476673 | C | G | 0.390 | 0.008 | 0.001 | 2.70E-21 |
| Testosterone | rs114949263 | 7 | 150498245 | C | T | 0.111 | 0.015 | 0.001 | 1.70E-34 |
| Testosterone | rs7808581 | 7 | 156194185 | C | T | 0.553 | 0.005 | 0.001 | 1.00E-09 |
| Testosterone | rs4240624 | 8 | 9184231 | A | G | 0.908 | 0.022 | 0.001 | 2.30E-59 |
| Testosterone | rs7464506 | 8 | 12624425 | A | T | 0.668 | 0.007 | 0.001 | 1.60E-13 |
| Testosterone | rs113973451 | 8 | 21906789 | G | T | 0.823 | 0.007 | 0.001 | 7.70E-13 |
| Testosterone | rs2241261 | 8 | 22876739 | C | T | 0.476 | 0.006 | 0.001 | 2.40E-14 |
| Testosterone | rs9644032 | 8 | 23414822 | T | G | 0.367 | 0.007 | 0.001 | 7.30E-18 |
| Testosterone | rs4739515 | 8 | 37391203 | C | G | 0.041 | 0.012 | 0.002 | 1.90E-08 |
| Testosterone | rs117318607 | 8 | 38947274 | G | A | 0.022 | 0.015 | 0.003 | 2.50E-09 |
| Testosterone | rs12543287 | 8 | 42334511 | C | G | 0.371 | 0.010 | 0.001 | 2.40E-37 |
| Testosterone | rs72656017 | 8 | 57152481 | A | G | 0.870 | 0.008 | 0.001 | 6.40E-12 |
| Testosterone | rs10504255 | 8 | 59398461 | A | G | 0.662 | 0.011 | 0.001 | 3.00E-36 |
| Testosterone | rs72663955 | 8 | 71139330 | T | G | 0.867 | 0.007 | 0.001 | 5.30E-09 |
| Testosterone | rs113605295 | 8 | 81374095 | T | C | 0.064 | 0.012 | 0.002 | 3.40E-14 |
| Testosterone | rs76767219 | 8 | 81426196 | A | C | 0.035 | 0.043 | 0.002 | 6.90E-83 |
| Testosterone | rs7828742 | 8 | 116960729 | A | G | 0.401 | 0.008 | 0.001 | 1.10E-20 |
| Testosterone | rs11774700 | 8 | 118220270 | C | T | 0.309 | 0.006 | 0.001 | 7.30E-13 |
| Testosterone | rs4871015 | 8 | 128314516 | A | G | 0.581 | 0.004 | 0.001 | 5.00E-08 |
| Testosterone | rs55831924 | 8 | 145031968 | T | C | 0.361 | 0.007 | 0.001 | 3.50E-15 |
| Testosterone | rs2721195 | 8 | 145677011 | T | C | 0.474 | 0.010 | 0.001 | 4.20E-33 |
| Testosterone | rs1567353 | 9 | 1033773 | C | G | 0.692 | 0.008 | 0.001 | 6.60E-18 |
| Testosterone | rs10757112 | 9 | 2011588 | A | G | 0.603 | 0.005 | 0.001 | 2.10E-08 |
| Testosterone | rs10114763 | 9 | 4143749 | T | A | 0.423 | 0.009 | 0.001 | 3.80E-26 |
| Testosterone | rs1330307 | 9 | 4305064 | A | C | 0.514 | 0.007 | 0.001 | 7.90E-18 |
| Testosterone | rs79793188 | 9 | 6449701 | C | T | 0.812 | 0.006 | 0.001 | 1.00E-08 |
| Testosterone | rs820504 | 9 | 6668278 | G | A | 0.864 | 0.012 | 0.001 | 1.70E-24 |
| Testosterone | rs2031316 | 9 | 13563624 | A | C | 0.081 | 0.010 | 0.002 | 4.50E-10 |
| Testosterone | rs10811662 | 9 | 22134253 | A | G | 0.173 | 0.007 | 0.001 | 1.10E-10 |
| Testosterone | rs35234337 | 9 | 35661243 | C | T | 0.742 | 0.006 | 0.001 | 3.30E-08 |
| Testosterone | rs10868080 | 9 | 86626769 | T | A | 0.256 | 0.023 | 0.001 | 3.40E-125 |
| Testosterone | rs143554698 | 9 | 95538573 | C | T | 0.859 | 0.008 | 0.001 | 1.50E-12 |
| Testosterone | rs1475543 | 9 | 100333671 | C | A | 0.827 | 0.006 | 0.001 | 1.40E-08 |
| Testosterone | rs11515536 | 9 | 101771183 | T | C | 0.131 | 0.007 | 0.001 | 1.60E-08 |
| Testosterone | rs4743776 | 9 | 107727727 | G | A | 0.299 | 0.006 | 0.001 | 1.30E-10 |
| Testosterone | rs62580766 | 9 | 113034490 | T | C | 0.182 | 0.011 | 0.001 | 3.20E-27 |
| Testosterone | rs6477768 | 9 | 113168239 | A | G | 0.682 | 0.006 | 0.001 | 2.50E-09 |
| Testosterone | rs17372936 | 9 | 119066203 | T | C | 0.768 | 0.008 | 0.001 | 4.10E-18 |
| Testosterone | rs4837794 | 9 | 123507855 | T | C | 0.331 | 0.008 | 0.001 | 1.70E-22 |
| Testosterone | rs700085 | 9 | 125870466 | T | C | 0.088 | 0.009 | 0.001 | 3.70E-11 |
| Testosterone | rs9697210 | 9 | 131468740 | G | A | 0.854 | 0.016 | 0.001 | 9.20E-45 |
| Testosterone | rs8176693 | 9 | 136137657 | C | T | 0.938 | 0.013 | 0.002 | 7.40E-14 |
| Testosterone | rs72766607 | 9 | 136895818 | T | G | 0.980 | 0.034 | 0.003 | 5.20E-32 |
| Testosterone | rs11791747 | 9 | 137106879 | G | A | 0.307 | 0.007 | 0.001 | 6.90E-15 |
| Testosterone | rs35233014 | 9 | 137268177 | C | A | 0.254 | 0.014 | 0.001 | 2.50E-46 |
| Testosterone | rs11103377 | 9 | 139097135 | G | A | 0.539 | 0.010 | 0.001 | 1.20E-32 |
| Testosterone | rs2279415 | 10 | 3792608 | G | A | 0.531 | 0.005 | 0.001 | 1.90E-08 |
| Testosterone | rs79717793 | 10 | 5262267 | G | A | 0.845 | 0.021 | 0.001 | 8.50E-84 |
| Testosterone | rs3824655 | 10 | 13370779 | C | G | 0.590 | 0.006 | 0.001 | 7.30E-14 |
| Testosterone | rs3737178 | 10 | 31607215 | A | G | 0.951 | 0.010 | 0.002 | 1.20E-08 |
| Testosterone | rs899865 | 10 | 36473044 | T | C | 0.600 | 0.005 | 0.001 | 1.20E-08 |
| Testosterone | rs72783094 | 10 | 49686734 | A | G | 0.908 | 0.008 | 0.001 | 1.80E-08 |
| Testosterone | rs1530439 | 10 | 63645959 | T | G | 0.309 | 0.009 | 0.001 | 7.90E-24 |
| Testosterone | rs541030121 | 10 | 64871859 | A | G | 0.065 | 0.016 | 0.002 | 5.50E-15 |
| Testosterone | rs537858427 | 10 | 65097399 | G | A | 0.100 | 0.010 | 0.002 | 1.50E-08 |
| Testosterone | rs10822163 | 10 | 65124098 | G | C | 0.473 | 0.053 | 0.001 | 1.00E-200 |
| Testosterone | rs1204083 | 10 | 69835197 | C | G | 0.346 | 0.007 | 0.001 | 2.50E-17 |
| Testosterone | rs7100001 | 10 | 74654754 | A | G | 0.049 | 0.011 | 0.002 | 1.30E-08 |
| Testosterone | rs2579162 | 10 | 79541639 | C | T | 0.709 | 0.004 | 0.001 | 1.50E-08 |
| Testosterone | rs1782652 | 10 | 81074125 | T | A | 0.619 | 0.012 | 0.001 | 2.90E-50 |
| Testosterone | rs11202594 | 10 | 89641222 | G | A | 0.843 | 0.006 | 0.001 | 9.40E-09 |
| Testosterone | rs1772189 | 10 | 93629499 | T | A | 0.523 | 0.012 | 0.001 | 9.40E-54 |
| Testosterone | rs2068888 | 10 | 94839642 | A | G | 0.450 | 0.011 | 0.001 | 1.70E-40 |
| Testosterone | rs7080472 | 10 | 96012950 | T | G | 0.423 | 0.005 | 0.001 | 1.30E-08 |
| Testosterone | rs11188601 | 10 | 97856899 | C | T | 0.364 | 0.008 | 0.001 | 1.20E-22 |
| Testosterone | rs2862954 | 10 | 101912064 | C | T | 0.500 | 0.010 | 0.001 | 3.30E-28 |
| Testosterone | rs67477288 | 10 | 103587858 | C | T | 0.942 | 0.010 | 0.002 | 2.70E-08 |
| Testosterone | rs80235628 | 10 | 122859270 | G | A | 0.950 | 0.018 | 0.002 | 2.30E-21 |
| Testosterone | rs7893136 | 10 | 122926460 | T | C | 0.021 | 0.018 | 0.003 | 5.50E-10 |
| Testosterone | rs4758639 | 11 | 305406 | A | G | 0.664 | 0.007 | 0.001 | 1.00E-13 |
| Testosterone | rs2412138 | 11 | 3090126 | T | C | 0.606 | 0.006 | 0.001 | 5.30E-13 |
| Testosterone | rs11601507 | 11 | 5701074 | A | C | 0.069 | 0.016 | 0.002 | 1.10E-25 |
| Testosterone | rs1037169 | 11 | 13361005 | T | C | 0.313 | 0.012 | 0.001 | 1.20E-41 |
| Testosterone | rs2074310 | 11 | 17421886 | C | T | 0.643 | 0.006 | 0.001 | 1.50E-12 |
| Testosterone | rs62618693 | 11 | 32956492 | T | C | 0.045 | 0.016 | 0.002 | 2.30E-16 |
| Testosterone | rs4756190 | 11 | 35124040 | T | C | 0.580 | 0.004 | 0.001 | 1.00E-08 |
| Testosterone | rs2292910 | 11 | 45903613 | A | C | 0.332 | 0.006 | 0.001 | 1.20E-11 |
| Testosterone | rs566217606 | 11 | 48877002 | C | T | 0.013 | 0.022 | 0.004 | 1.40E-08 |
| Testosterone | rs143709973 | 11 | 59572669 | A | C | 0.955 | 0.012 | 0.002 | 4.40E-10 |
| Testosterone | rs174533 | 11 | 61549025 | G | A | 0.652 | 0.010 | 0.001 | 1.20E-28 |
| Testosterone | rs12797706 | 11 | 65561369 | A | G | 0.235 | 0.014 | 0.001 | 3.20E-41 |
| Testosterone | rs4988308 | 11 | 68106842 | G | A | 0.557 | 0.005 | 0.001 | 1.20E-09 |
| Testosterone | rs631695 | 11 | 69283303 | T | G | 0.416 | 0.014 | 0.001 | 6.80E-68 |
| Testosterone | rs75713100 | 11 | 77517973 | T | G | 0.911 | 0.008 | 0.001 | 3.50E-09 |
| Testosterone | rs12575636 | 11 | 95311260 | T | G | 0.811 | 0.010 | 0.001 | 2.60E-18 |
| Testosterone | rs10895276 | 11 | 102083695 | C | T | 0.658 | 0.009 | 0.001 | 3.80E-25 |
| Testosterone | rs78312641 | 11 | 118749318 | A | T | 0.128 | 0.008 | 0.001 | 2.50E-10 |
| Testosterone | rs55771168 | 11 | 119070949 | C | T | 0.272 | 0.007 | 0.001 | 5.70E-15 |
| Testosterone | rs2156804 | 11 | 122610326 | G | T | 0.513 | 0.005 | 0.001 | 1.20E-09 |
| Testosterone | rs2369280 | 12 | 508859 | T | G | 0.737 | 0.005 | 0.001 | 3.00E-08 |
| Testosterone | rs56196860 | 12 | 2908330 | A | C | 0.031 | 0.023 | 0.002 | 4.40E-24 |
| Testosterone | rs76895963 | 12 | 4384844 | G | T | 0.021 | 0.073 | 0.003 | 1.20E-120 |
| Testosterone | rs3782735 | 12 | 6885076 | A | G | 0.600 | 0.008 | 0.001 | 1.20E-21 |
| Testosterone | rs11045171 | 12 | 20470199 | G | A | 0.198 | 0.008 | 0.001 | 1.30E-14 |
| Testosterone | rs57743625 | 12 | 21367633 | G | A | 0.840 | 0.030 | 0.001 | 1.10E-159 |
| Testosterone | rs2900528 | 12 | 24150441 | C | G | 0.417 | 0.006 | 0.001 | 1.50E-12 |
| Testosterone | rs1391790 | 12 | 24203096 | T | C | 0.964 | 0.025 | 0.002 | 1.80E-30 |
| Testosterone | rs75130744 | 12 | 25410741 | G | C | 0.929 | 0.027 | 0.002 | 7.30E-72 |
| Testosterone | rs2129869 | 12 | 26457650 | A | T | 0.780 | 0.007 | 0.001 | 1.20E-12 |
| Testosterone | rs34072608 | 12 | 46189284 | G | T | 0.801 | 0.007 | 0.001 | 1.60E-13 |
| Testosterone | rs118080406 | 12 | 47249428 | G | A | 0.025 | 0.015 | 0.003 | 2.30E-08 |
| Testosterone | rs56365029 | 12 | 49036347 | A | G | 0.978 | 0.017 | 0.003 | 1.00E-10 |
| Testosterone | rs864899 | 12 | 51221127 | A | G | 0.415 | 0.012 | 0.001 | 5.20E-47 |
| Testosterone | rs12818938 | 12 | 53783182 | T | G | 0.832 | 0.009 | 0.001 | 4.10E-14 |
| Testosterone | rs73139029 | 12 | 62822406 | A | T | 0.859 | 0.007 | 0.001 | 2.40E-09 |
| Testosterone | rs145775785 | 12 | 65902265 | T | C | 0.015 | 0.023 | 0.004 | 6.10E-10 |
| Testosterone | rs2583939 | 12 | 66213521 | C | T | 0.868 | 0.008 | 0.001 | 6.40E-10 |
| Testosterone | rs1042725 | 12 | 66358347 | C | T | 0.508 | 0.008 | 0.001 | 7.50E-25 |
| Testosterone | rs11176664 | 12 | 67661519 | A | G | 0.495 | 0.005 | 0.001 | 3.40E-09 |
| Testosterone | rs2601007 | 12 | 69979115 | G | C | 0.654 | 0.005 | 0.001 | 2.70E-10 |
| Testosterone | rs374335 | 12 | 77457439 | A | G | 0.681 | 0.006 | 0.001 | 2.80E-10 |
| Testosterone | rs11107124 | 12 | 93988283 | G | C | 0.711 | 0.006 | 0.001 | 2.50E-10 |
| Testosterone | rs11108061 | 12 | 95857620 | T | C | 0.427 | 0.005 | 0.001 | 8.70E-09 |
| Testosterone | rs11111274 | 12 | 102838128 | G | A | 0.263 | 0.010 | 0.001 | 2.50E-25 |
| Testosterone | rs112725417 | 12 | 112657361 | T | C | 0.973 | 0.017 | 0.003 | 3.50E-11 |
| Testosterone | rs4767327 | 12 | 115929191 | T | A | 0.618 | 0.005 | 0.001 | 7.90E-10 |
| Testosterone | rs113257350 | 12 | 120381998 | C | T | 0.978 | 0.017 | 0.003 | 4.60E-10 |
| Testosterone | rs2393775 | 12 | 121424574 | A | G | 0.622 | 0.015 | 0.001 | 2.00E-80 |
| Testosterone | rs73214164 | 12 | 121470978 | T | C | 0.802 | 0.010 | 0.001 | 4.90E-22 |
| Testosterone | rs12311848 | 12 | 124486851 | G | A | 0.334 | 0.010 | 0.001 | 1.10E-33 |
| Testosterone | rs1725788 | 12 | 131608476 | G | A | 0.238 | 0.006 | 0.001 | 3.30E-09 |
| Testosterone | rs749170 | 13 | 22350875 | T | C | 0.335 | 0.005 | 0.001 | 7.00E-10 |
| Testosterone | rs9533843 | 13 | 44980150 | A | G | 0.466 | 0.005 | 0.001 | 1.80E-08 |
| Testosterone | rs41284816 | 13 | 50655989 | T | G | 0.019 | 0.031 | 0.003 | 1.50E-25 |
| Testosterone | rs3116625 | 13 | 50945251 | C | T | 0.800 | 0.007 | 0.001 | 1.10E-13 |
| Testosterone | rs11843816 | 13 | 91987065 | C | T | 0.965 | 0.013 | 0.002 | 3.00E-08 |
| Testosterone | rs750598 | 13 | 111028978 | A | G | 0.338 | 0.005 | 0.001 | 9.50E-09 |
| Testosterone | rs373373 | 13 | 111297952 | A | C | 0.948 | 0.011 | 0.002 | 1.30E-09 |
| Testosterone | rs116338429 | 13 | 114767040 | T | C | 0.171 | 0.008 | 0.001 | 2.10E-11 |
| Testosterone | rs112035922 | 13 | 115047464 | C | T | 0.768 | 0.008 | 0.001 | 1.90E-18 |
| Testosterone | rs2064482 | 14 | 23709315 | T | C | 0.255 | 0.012 | 0.001 | 1.50E-43 |
| Testosterone | rs11621792 | 14 | 24871926 | C | T | 0.547 | 0.019 | 0.001 | 1.80E-117 |
| Testosterone | rs12435790 | 14 | 35154381 | G | A | 0.078 | 0.010 | 0.002 | 5.80E-10 |
| Testosterone | rs2239222 | 14 | 73011885 | G | A | 0.349 | 0.011 | 0.001 | 9.40E-35 |
| Testosterone | rs13379043 | 14 | 74250126 | C | T | 0.279 | 0.010 | 0.001 | 2.60E-28 |
| Testosterone | rs1005421 | 14 | 89886940 | C | T | 0.584 | 0.007 | 0.001 | 7.60E-20 |
| Testosterone | rs28929474 | 14 | 94844947 | T | C | 0.020 | 0.096 | 0.003 | 1.00E-200 |
| Testosterone | rs17580 | 14 | 94847262 | A | T | 0.048 | 0.026 | 0.002 | 1.90E-42 |
| Testosterone | rs3742366 | 14 | 104198351 | C | T | 0.346 | 0.008 | 0.001 | 9.70E-23 |
| Testosterone | rs2498786 | 14 | 105262368 | C | G | 0.384 | 0.010 | 0.001 | 1.50E-32 |
| Testosterone | rs56112295 | 14 | 105877057 | T | C | 0.226 | 0.006 | 0.001 | 1.60E-10 |
| Testosterone | rs28510484 | 15 | 31637569 | G | C | 0.829 | 0.008 | 0.001 | 3.80E-12 |
| Testosterone | rs55800572 | 15 | 35285183 | G | C | 0.339 | 0.008 | 0.001 | 6.80E-20 |
| Testosterone | rs2454352 | 15 | 36047091 | C | T | 0.239 | 0.007 | 0.001 | 1.80E-12 |
| Testosterone | rs275177 | 15 | 39449003 | C | T | 0.149 | 0.008 | 0.001 | 2.70E-11 |
| Testosterone | rs28790585 | 15 | 39668870 | C | T | 0.705 | 0.006 | 0.001 | 1.50E-09 |
| Testosterone | rs11637681 | 15 | 40387971 | A | G | 0.723 | 0.008 | 0.001 | 2.20E-18 |
| Testosterone | rs139974673 | 15 | 44027885 | T | C | 0.975 | 0.066 | 0.003 | 5.10E-147 |
| Testosterone | rs1008805 | 15 | 51549599 | A | G | 0.576 | 0.005 | 0.001 | 6.10E-09 |
| Testosterone | rs181598957 | 15 | 53327792 | A | G | 0.018 | 0.024 | 0.003 | 3.50E-13 |
| Testosterone | rs79391862 | 15 | 53739426 | A | C | 0.986 | 0.082 | 0.004 | 2.20E-121 |
| Testosterone | rs4775191 | 15 | 59926946 | A | C | 0.339 | 0.007 | 0.001 | 9.80E-16 |
| Testosterone | rs339998 | 15 | 60947763 | T | C | 0.394 | 0.005 | 0.001 | 5.10E-09 |
| Testosterone | rs3848125 | 15 | 61957236 | A | G | 0.577 | 0.005 | 0.001 | 1.50E-11 |
| Testosterone | rs76428668 | 15 | 66839282 | T | G | 0.750 | 0.009 | 0.001 | 9.10E-24 |
| Testosterone | rs8038465 | 15 | 73978337 | T | C | 0.425 | 0.005 | 0.001 | 3.00E-10 |
| Testosterone | rs112500920 | 15 | 82507605 | T | C | 0.930 | 0.012 | 0.002 | 4.00E-11 |
| Testosterone | rs9672839 | 15 | 93568884 | C | A | 0.395 | 0.005 | 0.001 | 6.50E-09 |
| Testosterone | rs12898856 | 15 | 96227920 | C | T | 0.551 | 0.010 | 0.001 | 5.40E-36 |
| Testosterone | rs56332871 | 15 | 96714816 | A | C | 0.272 | 0.035 | 0.001 | 1.00E-200 |
| Testosterone | rs28469124 | 16 | 1842973 | C | G | 0.087 | 0.010 | 0.001 | 6.60E-13 |
| Testosterone | rs28372698 | 16 | 3115111 | A | T | 0.406 | 0.005 | 0.001 | 6.00E-09 |
| Testosterone | rs3747587 | 16 | 4674954 | G | C | 0.814 | 0.010 | 0.001 | 3.00E-21 |
| Testosterone | rs720130 | 16 | 11132633 | G | T | 0.595 | 0.005 | 0.001 | 5.10E-12 |
| Testosterone | rs3743588 | 16 | 11836508 | G | A | 0.715 | 0.007 | 0.001 | 9.00E-17 |
| Testosterone | rs12928099 | 16 | 15150505 | A | C | 0.295 | 0.010 | 0.001 | 4.00E-29 |
| Testosterone | rs41278174 | 16 | 16259596 | A | G | 0.027 | 0.013 | 0.003 | 3.00E-08 |
| Testosterone | rs34050011 | 16 | 49868513 | A | C | 0.424 | 0.006 | 0.001 | 1.20E-09 |
| Testosterone | rs11643656 | 16 | 51463539 | G | A | 0.825 | 0.007 | 0.001 | 5.40E-09 |
| Testosterone | rs246192 | 16 | 58544295 | G | C | 0.479 | 0.007 | 0.001 | 1.40E-16 |
| Testosterone | rs61733486 | 16 | 68390697 | C | T | 0.942 | 0.011 | 0.002 | 5.60E-10 |
| Testosterone | rs77147683 | 16 | 71623594 | A | C | 0.233 | 0.005 | 0.001 | 8.70E-09 |
| Testosterone | rs2925979 | 16 | 81534790 | C | T | 0.699 | 0.007 | 0.001 | 2.30E-16 |
| Testosterone | rs4782568 | 16 | 83980529 | G | C | 0.452 | 0.013 | 0.001 | 4.30E-58 |
| Testosterone | rs11641834 | 16 | 88070573 | C | T | 0.569 | 0.010 | 0.001 | 1.00E-36 |
| Testosterone | rs56292801 | 16 | 88535341 | A | G | 0.273 | 0.009 | 0.001 | 1.00E-25 |
| Testosterone | rs11078597 | 17 | 1618363 | C | T | 0.186 | 0.017 | 0.001 | 2.70E-61 |
| Testosterone | rs9902384 | 17 | 5171746 | A | G | 0.753 | 0.006 | 0.001 | 8.20E-09 |
| Testosterone | rs858519 | 17 | 7531965 | C | T | 0.557 | 0.097 | 0.001 | 1.00E-200 |
| Testosterone | rs8066941 | 17 | 9588450 | T | G | 0.762 | 0.012 | 0.001 | 5.90E-40 |
| Testosterone | rs17669311 | 17 | 13837051 | G | A | 0.611 | 0.009 | 0.001 | 2.10E-29 |
| Testosterone | rs12937088 | 17 | 16031029 | G | A | 0.508 | 0.006 | 0.001 | 8.50E-17 |
| Testosterone | rs8079418 | 17 | 17924060 | C | T | 0.612 | 0.014 | 0.001 | 3.70E-65 |
| Testosterone | rs12943365 | 17 | 29680526 | G | C | 0.609 | 0.009 | 0.001 | 5.50E-30 |
| Testosterone | rs17138478 | 17 | 36073320 | A | C | 0.129 | 0.009 | 0.001 | 3.30E-14 |
| Testosterone | rs17616365 | 17 | 38256401 | G | A | 0.968 | 0.022 | 0.002 | 3.20E-23 |
| Testosterone | rs650558 | 17 | 40721042 | C | T | 0.751 | 0.007 | 0.001 | 2.20E-14 |
| Testosterone | rs10048173 | 17 | 45592266 | G | A | 0.513 | 0.015 | 0.001 | 1.50E-80 |
| Testosterone | rs11655704 | 17 | 47448172 | C | T | 0.314 | 0.032 | 0.001 | 1.00E-200 |
| Testosterone | rs8077316 | 17 | 48626389 | T | C | 0.246 | 0.006 | 0.001 | 1.60E-10 |
| Testosterone | rs8074363 | 17 | 59241469 | C | T | 0.775 | 0.006 | 0.001 | 6.10E-11 |
| Testosterone | rs76708468 | 17 | 62206299 | T | C | 0.961 | 0.013 | 0.002 | 4.50E-09 |
| Testosterone | rs17650301 | 17 | 62479273 | C | A | 0.294 | 0.005 | 0.001 | 1.20E-09 |
| Testosterone | rs1801689 | 17 | 64210580 | A | C | 0.970 | 0.038 | 0.002 | 3.00E-60 |
| Testosterone | rs7211695 | 17 | 65235971 | A | G | 0.521 | 0.008 | 0.001 | 1.40E-24 |
| Testosterone | rs34931250 | 17 | 66879927 | T | C | 0.061 | 0.010 | 0.002 | 6.10E-10 |
| Testosterone | rs9282552 | 17 | 67109894 | T | C | 0.650 | 0.007 | 0.001 | 4.70E-18 |
| Testosterone | rs1605750 | 17 | 68471073 | A | G | 0.510 | 0.005 | 0.001 | 4.60E-10 |
| Testosterone | rs72844546 | 17 | 73149850 | C | T | 0.346 | 0.010 | 0.001 | 2.80E-33 |
| Testosterone | rs2587505 | 17 | 77784268 | T | C | 0.580 | 0.006 | 0.001 | 1.30E-11 |
| Testosterone | rs10153315 | 17 | 79481772 | T | C | 0.582 | 0.008 | 0.001 | 2.90E-24 |
| Testosterone | rs11664106 | 18 | 2846812 | T | A | 0.373 | 0.007 | 0.001 | 7.10E-17 |
| Testosterone | rs9945126 | 18 | 46469962 | C | G | 0.465 | 0.004 | 0.001 | 1.30E-08 |
| Testosterone | rs55855238 | 18 | 55089715 | C | T | 0.650 | 0.010 | 0.001 | 5.10E-29 |
| Testosterone | rs6567230 | 18 | 59330824 | T | A | 0.701 | 0.006 | 0.001 | 1.60E-11 |
| Testosterone | rs620068 | 18 | 60140576 | A | G | 0.459 | 0.005 | 0.001 | 7.60E-10 |
| Testosterone | rs12454712 | 18 | 60845884 | C | T | 0.377 | 0.010 | 0.001 | 1.00E-32 |
| Testosterone | rs3829639 | 18 | 71943144 | A | G | 0.672 | 0.008 | 0.001 | 1.30E-19 |
| Testosterone | rs34668346 | 19 | 672169 | A | G | 0.513 | 0.005 | 0.001 | 8.90E-10 |
| Testosterone | rs150122016 | 19 | 1246079 | G | T | 0.972 | 0.016 | 0.003 | 8.10E-09 |
| Testosterone | rs1640269 | 19 | 2793194 | C | A | 0.286 | 0.019 | 0.001 | 9.10E-101 |
| Testosterone | rs11539938 | 19 | 3062857 | C | T | 0.421 | 0.010 | 0.001 | 2.40E-29 |
| Testosterone | rs60018147 | 19 | 3375572 | G | A | 0.120 | 0.014 | 0.001 | 2.60E-25 |
| Testosterone | rs10221473 | 19 | 7236626 | G | A | 0.542 | 0.007 | 0.001 | 6.60E-13 |
| Testosterone | rs8107967 | 19 | 7972615 | G | A | 0.567 | 0.007 | 0.001 | 1.30E-15 |
| Testosterone | rs281439 | 19 | 10400110 | C | G | 0.780 | 0.006 | 0.001 | 3.00E-08 |
| Testosterone | rs8101895 | 19 | 12508061 | A | T | 0.236 | 0.007 | 0.001 | 3.50E-13 |
| Testosterone | rs7252372 | 19 | 14172896 | G | C | 0.556 | 0.009 | 0.001 | 8.20E-27 |
| Testosterone | rs202200760 | 19 | 17346854 | C | G | 0.039 | 0.073 | 0.002 | 1.00E-200 |
| Testosterone | rs4805881 | 19 | 33896432 | C | A | 0.666 | 0.009 | 0.001 | 4.30E-29 |
| Testosterone | rs45512696 | 19 | 35550878 | T | C | 0.175 | 0.021 | 0.001 | 2.00E-86 |
| Testosterone | rs11666245 | 19 | 38229926 | G | A | 0.953 | 0.017 | 0.002 | 7.00E-20 |
| Testosterone | rs11673023 | 19 | 38239521 | C | A | 0.829 | 0.007 | 0.001 | 9.60E-12 |
| Testosterone | rs483082 | 19 | 45416178 | T | G | 0.237 | 0.009 | 0.001 | 1.90E-21 |
| Testosterone | rs5112 | 19 | 45430280 | G | C | 0.533 | 0.008 | 0.001 | 4.90E-20 |
| Testosterone | rs34255979 | 19 | 46384830 | T | C | 0.120 | 0.028 | 0.001 | 1.50E-108 |
| Testosterone | rs111981233 | 19 | 50016479 | G | T | 0.080 | 0.021 | 0.002 | 1.60E-46 |
| Testosterone | rs11672485 | 19 | 53833712 | C | T | 0.490 | 0.005 | 0.001 | 1.10E-12 |
| Testosterone | rs4077285 | 19 | 56599405 | G | C | 0.906 | 0.012 | 0.001 | 1.90E-15 |
| Testosterone | rs16988208 | 19 | 58332315 | G | T | 0.164 | 0.007 | 0.001 | 1.70E-10 |
| Testosterone | rs11545185 | 19 | 59028585 | A | G | 0.171 | 0.009 | 0.001 | 2.20E-16 |
| Testosterone | rs144033177 | 20 | 571467 | A | C | 0.984 | 0.018 | 0.003 | 1.10E-08 |
| Testosterone | rs1741288 | 20 | 4102954 | A | G | 0.635 | 0.005 | 0.001 | 2.30E-11 |
| Testosterone | rs7261425 | 20 | 20068635 | G | C | 0.278 | 0.005 | 0.001 | 2.70E-08 |
| Testosterone | rs6048205 | 20 | 22559601 | A | G | 0.957 | 0.012 | 0.002 | 3.60E-08 |
| Testosterone | rs13042148 | 20 | 32298286 | C | T | 0.845 | 0.014 | 0.001 | 9.00E-39 |
| Testosterone | rs6029640 | 20 | 39970385 | G | A | 0.420 | 0.010 | 0.001 | 9.60E-33 |
| Testosterone | rs6073431 | 20 | 43040569 | T | C | 0.531 | 0.017 | 0.001 | 5.10E-92 |
| Testosterone | rs1412957 | 20 | 45557065 | A | G | 0.574 | 0.007 | 0.001 | 3.50E-16 |
| Testosterone | rs6018424 | 20 | 45986984 | T | C | 0.199 | 0.008 | 0.001 | 4.40E-14 |
| Testosterone | rs55987409 | 20 | 49569025 | T | C | 0.072 | 0.018 | 0.002 | 6.40E-28 |
| Testosterone | rs61744628 | 20 | 52186837 | A | G | 0.027 | 0.017 | 0.003 | 1.70E-11 |
| Testosterone | rs6123685 | 20 | 55836040 | A | G | 0.254 | 0.005 | 0.001 | 3.80E-09 |
| Testosterone | rs62217799 | 20 | 62347191 | G | T | 0.342 | 0.007 | 0.001 | 2.20E-15 |
| Testosterone | rs1475883 | 21 | 17591913 | G | A | 0.567 | 0.004 | 0.001 | 2.40E-08 |
| Testosterone | rs112078975 | 21 | 33095959 | G | A | 0.951 | 0.012 | 0.002 | 8.40E-11 |
| Testosterone | rs4818008 | 21 | 40611442 | T | A | 0.645 | 0.005 | 0.001 | 2.70E-08 |
| Testosterone | rs35598889 | 22 | 18450794 | C | T | 0.233 | 0.007 | 0.001 | 8.50E-15 |
| Testosterone | rs759404 | 22 | 18916180 | C | T | 0.932 | 0.010 | 0.002 | 4.00E-08 |
| Testosterone | rs9606233 | 22 | 20066611 | C | G | 0.405 | 0.005 | 0.001 | 8.90E-12 |
| Testosterone | rs4820091 | 22 | 21940189 | G | T | 0.181 | 0.011 | 0.001 | 1.60E-21 |
| Testosterone | rs6005840 | 22 | 29101357 | A | G | 0.326 | 0.012 | 0.001 | 3.00E-42 |
| Testosterone | rs5749082 | 22 | 30770603 | T | A | 0.292 | 0.011 | 0.001 | 3.10E-36 |
| Testosterone | rs9610329 | 22 | 36042986 | C | T | 0.572 | 0.006 | 0.001 | 7.60E-13 |
| Testosterone | rs13057133 | 22 | 38179473 | C | T | 0.699 | 0.007 | 0.001 | 3.00E-15 |
| Testosterone | rs2075915 | 22 | 39254556 | A | G | 0.747 | 0.006 | 0.001 | 2.40E-11 |
| Testosterone | rs738409 | 22 | 44324727 | G | C | 0.216 | 0.023 | 0.001 | 3.10E-114 |
| Testosterone | rs135563 | 22 | 46536017 | T | C | 0.510 | 0.005 | 0.001 | 1.70E-09 |
| Testosterone | rs36171610 | 22 | 50422385 | G | A | 0.509 | 0.006 | 0.001 | 2.00E-11 |
| SHBG | rs7539725 | 1 | 10125407 | G | A | 0.137 | 0.007 | 0.001 | 8.00E-10 |
| SHBG | rs75077113 | 1 | 11214582 | C | A | 0.277 | 0.009 | 0.001 | 2.40E-22 |
| SHBG | rs198358 | 1 | 11904076 | C | T | 0.248 | 0.007 | 0.001 | 6.90E-13 |
| SHBG | rs36086195 | 1 | 16510894 | T | C | 0.580 | 0.011 | 0.001 | 2.90E-43 |
| SHBG | rs114165349 | 1 | 27021913 | G | C | 0.977 | 0.081 | 0.003 | 1.00E-200 |
| SHBG | rs111642750 | 1 | 29320013 | A | G | 0.051 | 0.011 | 0.002 | 8.10E-10 |
| SHBG | rs947643 | 1 | 31461438 | G | A | 0.752 | 0.005 | 0.001 | 2.30E-08 |
| SHBG | rs1969213 | 1 | 42238531 | A | G | 0.528 | 0.005 | 0.001 | 3.40E-11 |
| SHBG | rs2782640 | 1 | 44009033 | T | C | 0.622 | 0.005 | 0.001 | 3.50E-09 |
| SHBG | rs1883783 | 1 | 54890956 | T | G | 0.430 | 0.006 | 0.001 | 6.50E-14 |
| SHBG | rs35067979 | 1 | 61925076 | C | T | 0.929 | 0.015 | 0.002 | 2.20E-17 |
| SHBG | rs469864 | 1 | 91542517 | T | C | 0.791 | 0.009 | 0.001 | 6.40E-17 |
| SHBG | rs12385720 | 1 | 93539383 | G | A | 0.371 | 0.007 | 0.001 | 6.60E-19 |
| SHBG | rs1730859 | 1 | 107617707 | G | A | 0.343 | 0.026 | 0.001 | 6.60E-197 |
| SHBG | rs140584594 | 1 | 110232983 | A | G | 0.270 | 0.013 | 0.001 | 3.60E-49 |
| SHBG | rs41264630 | 1 | 149885800 | A | G | 0.080 | 0.013 | 0.002 | 7.20E-20 |
| SHBG | rs267733 | 1 | 150958836 | A | G | 0.839 | 0.012 | 0.001 | 6.60E-29 |
| SHBG | rs72694845 | 1 | 151909812 | A | C | 0.970 | 0.017 | 0.002 | 2.10E-12 |
| SHBG | rs9427104 | 1 | 154589232 | T | C | 0.479 | 0.012 | 0.001 | 8.60E-53 |
| SHBG | rs10797877 | 1 | 171075556 | C | T | 0.529 | 0.006 | 0.001 | 1.20E-15 |
| SHBG | rs12138803 | 1 | 172348823 | C | T | 0.730 | 0.006 | 0.001 | 2.30E-10 |
| SHBG | rs2274432 | 1 | 184020945 | G | A | 0.653 | 0.005 | 0.001 | 2.50E-11 |
| SHBG | rs78444298 | 1 | 184672098 | G | A | 0.980 | 0.025 | 0.003 | 1.80E-18 |
| SHBG | rs4639796 | 1 | 197126649 | G | A | 0.840 | 0.012 | 0.001 | 4.30E-28 |
| SHBG | rs17583875 | 1 | 197924770 | A | G | 0.021 | 0.027 | 0.003 | 2.70E-23 |
| SHBG | rs7540115 | 1 | 200265618 | C | A | 0.819 | 0.007 | 0.001 | 6.00E-12 |
| SHBG | rs2802770 | 1 | 203518456 | A | T | 0.537 | 0.007 | 0.001 | 5.90E-15 |
| SHBG | rs2369633 | 1 | 205181062 | T | C | 0.092 | 0.011 | 0.001 | 3.50E-15 |
| SHBG | rs1418652 | 1 | 205646458 | C | T | 0.386 | 0.006 | 0.001 | 6.50E-12 |
| SHBG | rs2456827 | 1 | 212942595 | T | C | 0.786 | 0.005 | 0.001 | 2.00E-08 |
| SHBG | rs1223791 | 1 | 214321081 | G | A | 0.163 | 0.015 | 0.001 | 3.30E-44 |
| SHBG | rs7539006 | 1 | 214398058 | A | C | 0.255 | 0.006 | 0.001 | 1.60E-11 |
| SHBG | rs3001032 | 1 | 219727779 | C | T | 0.320 | 0.011 | 0.001 | 1.30E-37 |
| SHBG | rs2247213 | 1 | 221055463 | G | A | 0.670 | 0.012 | 0.001 | 1.10E-45 |
| SHBG | rs2234922 | 1 | 226026406 | A | G | 0.800 | 0.008 | 0.001 | 6.80E-13 |
| SHBG | rs1870927 | 1 | 226426337 | A | T | 0.621 | 0.007 | 0.001 | 3.70E-18 |
| SHBG | rs3887753 | 1 | 227695517 | C | T | 0.177 | 0.007 | 0.001 | 2.60E-11 |
| SHBG | rs13402475 | 2 | 3639909 | C | G | 0.185 | 0.006 | 0.001 | 3.00E-08 |
| SHBG | rs4668732 | 2 | 11716919 | T | A | 0.637 | 0.006 | 0.001 | 2.90E-13 |
| SHBG | rs11096542 | 2 | 18707873 | G | A | 0.407 | 0.005 | 0.001 | 5.40E-11 |
| SHBG | rs35633876 | 2 | 20363074 | G | T | 0.516 | 0.008 | 0.001 | 2.90E-23 |
| SHBG | rs62130499 | 2 | 27184873 | A | C | 0.088 | 0.010 | 0.001 | 6.70E-12 |
| SHBG | rs4665972 | 2 | 27598097 | C | T | 0.607 | 0.037 | 0.001 | 1.00E-200 |
| SHBG | rs10210970 | 2 | 28646847 | C | T | 0.871 | 0.009 | 0.001 | 7.90E-15 |
| SHBG | rs72798731 | 2 | 32515337 | T | C | 0.034 | 0.014 | 0.002 | 7.20E-09 |
| SHBG | rs56219475 | 2 | 39241107 | A | G | 0.009 | 0.026 | 0.005 | 1.00E-09 |
| SHBG | rs6736913 | 2 | 42510018 | A | G | 0.021 | 0.032 | 0.003 | 2.30E-32 |
| SHBG | rs11690748 | 2 | 48584575 | C | G | 0.623 | 0.006 | 0.001 | 7.30E-15 |
| SHBG | rs998230 | 2 | 55121930 | C | G | 0.608 | 0.005 | 0.001 | 2.90E-09 |
| SHBG | rs17008851 | 2 | 61606097 | G | A | 0.133 | 0.010 | 0.001 | 2.10E-16 |
| SHBG | rs6546096 | 2 | 64906295 | A | G | 0.262 | 0.021 | 0.001 | 3.00E-110 |
| SHBG | rs11902527 | 2 | 66210213 | A | G | 0.676 | 0.005 | 0.001 | 2.00E-08 |
| SHBG | rs12624244 | 2 | 70417138 | A | G | 0.936 | 0.020 | 0.002 | 7.60E-33 |
| SHBG | rs2670747 | 2 | 71525784 | G | A | 0.127 | 0.007 | 0.001 | 2.50E-10 |
| SHBG | rs11164095 | 2 | 97155208 | T | C | 0.279 | 0.006 | 0.001 | 4.10E-10 |
| SHBG | rs3747647 | 2 | 112245586 | C | G | 0.218 | 0.009 | 0.001 | 3.70E-19 |
| SHBG | rs3979376 | 2 | 113225155 | A | G | 0.475 | 0.004 | 0.001 | 1.30E-09 |
| SHBG | rs10211038 | 2 | 114593805 | A | G | 0.189 | 0.007 | 0.001 | 2.30E-10 |
| SHBG | rs11688682 | 2 | 121347612 | C | G | 0.271 | 0.007 | 0.001 | 1.90E-16 |
| SHBG | rs2307394 | 2 | 148716428 | C | T | 0.303 | 0.006 | 0.001 | 7.10E-12 |
| SHBG | rs13389219 | 2 | 165528876 | T | C | 0.393 | 0.016 | 0.001 | 1.60E-88 |
| SHBG | rs10187560 | 2 | 173925051 | T | C | 0.303 | 0.005 | 0.001 | 8.90E-10 |
| SHBG | rs2364717 | 2 | 178101235 | T | C | 0.539 | 0.006 | 0.001 | 3.30E-16 |
| SHBG | rs10169561 | 2 | 180458771 | C | T | 0.464 | 0.005 | 0.001 | 6.30E-09 |
| SHBG | rs1047891 | 2 | 211540507 | A | C | 0.316 | 0.010 | 0.001 | 1.20E-29 |
| SHBG | rs2014998 | 2 | 217681961 | A | G | 0.260 | 0.005 | 0.001 | 3.20E-08 |
| SHBG | rs62182125 | 2 | 219274142 | G | A | 0.449 | 0.009 | 0.001 | 1.50E-25 |
| SHBG | rs78058190 | 2 | 219699999 | G | A | 0.950 | 0.021 | 0.002 | 2.00E-24 |
| SHBG | rs57467915 | 2 | 220081416 | G | A | 0.985 | 0.022 | 0.003 | 3.90E-11 |
| SHBG | rs4674669 | 2 | 223430985 | C | T | 0.139 | 0.007 | 0.001 | 9.10E-09 |
| SHBG | rs2943641 | 2 | 227093745 | T | C | 0.353 | 0.014 | 0.001 | 9.80E-66 |
| SHBG | rs11682084 | 2 | 231286503 | C | A | 0.714 | 0.006 | 0.001 | 3.20E-13 |
| SHBG | rs62195072 | 2 | 234260879 | C | T | 0.324 | 0.008 | 0.001 | 3.30E-18 |
| SHBG | rs28898590 | 2 | 234619422 | G | T | 0.934 | 0.011 | 0.002 | 5.20E-11 |
| SHBG | rs62193162 | 2 | 242268436 | T | A | 0.639 | 0.005 | 0.001 | 3.30E-09 |
| SHBG | rs1801282 | 3 | 12393125 | G | C | 0.120 | 0.022 | 0.001 | 3.00E-67 |
| SHBG | rs6792725 | 3 | 24520283 | G | A | 0.693 | 0.015 | 0.001 | 1.10E-63 |
| SHBG | rs784504 | 3 | 39195260 | C | G | 0.811 | 0.007 | 0.001 | 4.00E-14 |
| SHBG | rs12487736 | 3 | 47459679 | C | T | 0.424 | 0.009 | 0.001 | 3.90E-27 |
| SHBG | rs1965132 | 3 | 69147519 | C | A | 0.505 | 0.005 | 0.001 | 1.90E-10 |
| SHBG | rs2597305 | 3 | 70922562 | G | C | 0.454 | 0.005 | 0.001 | 7.30E-10 |
| SHBG | rs13082048 | 3 | 86964322 | A | G | 0.361 | 0.006 | 0.001 | 2.60E-10 |
| SHBG | rs958650 | 3 | 98736153 | G | A | 0.770 | 0.006 | 0.001 | 6.90E-09 |
| SHBG | rs13315174 | 3 | 105406468 | G | A | 0.784 | 0.008 | 0.001 | 6.00E-17 |
| SHBG | rs6776396 | 3 | 124321704 | T | C | 0.246 | 0.005 | 0.001 | 4.50E-08 |
| SHBG | rs2953761 | 3 | 129745975 | A | G | 0.776 | 0.006 | 0.001 | 3.30E-10 |
| SHBG | rs62292950 | 3 | 132197995 | T | G | 0.868 | 0.006 | 0.001 | 1.80E-08 |
| SHBG | rs687339 | 3 | 135932359 | C | T | 0.228 | 0.027 | 0.001 | 1.30E-175 |
| SHBG | rs6777420 | 3 | 142002869 | T | C | 0.131 | 0.007 | 0.001 | 8.80E-09 |
| SHBG | rs9834503 | 3 | 149994882 | A | C | 0.539 | 0.006 | 0.001 | 1.70E-11 |
| SHBG | rs62271373 | 3 | 150066540 | T | A | 0.940 | 0.018 | 0.002 | 2.30E-24 |
| SHBG | rs55735727 | 3 | 169488148 | T | A | 0.269 | 0.007 | 0.001 | 2.60E-13 |
| SHBG | rs10936702 | 3 | 171548897 | A | G | 0.394 | 0.005 | 0.001 | 7.90E-09 |
| SHBG | rs79287178 | 3 | 172294500 | G | A | 0.969 | 0.037 | 0.003 | 1.70E-51 |
| SHBG | rs234043 | 3 | 172313367 | T | C | 0.282 | 0.009 | 0.001 | 1.10E-27 |
| SHBG | rs2293606 | 3 | 184106493 | G | T | 0.056 | 0.011 | 0.002 | 7.50E-10 |
| SHBG | rs57158761 | 3 | 185371172 | A | G | 0.564 | 0.009 | 0.001 | 1.70E-27 |
| SHBG | rs7430950 | 3 | 196233136 | A | C | 0.747 | 0.006 | 0.001 | 3.60E-11 |
| SHBG | rs11725653 | 4 | 963397 | C | T | 0.894 | 0.011 | 0.001 | 2.90E-17 |
| SHBG | rs13108218 | 4 | 3443931 | A | G | 0.383 | 0.024 | 0.001 | 3.00E-172 |
| SHBG | rs4450871 | 4 | 4990298 | G | A | 0.442 | 0.006 | 0.001 | 6.40E-14 |
| SHBG | rs2724475 | 4 | 17946432 | T | C | 0.262 | 0.007 | 0.001 | 5.20E-14 |
| SHBG | rs2970877 | 4 | 23887454 | G | T | 0.297 | 0.009 | 0.001 | 1.50E-20 |
| SHBG | rs28473232 | 4 | 37406477 | G | A | 0.232 | 0.006 | 0.001 | 2.60E-09 |
| SHBG | rs2381145 | 4 | 38452427 | C | T | 0.799 | 0.006 | 0.001 | 2.70E-08 |
| SHBG | rs6531735 | 4 | 39686332 | G | A | 0.493 | 0.005 | 0.001 | 1.00E-09 |
| SHBG | rs7696472 | 4 | 69538180 | A | G | 0.475 | 0.013 | 0.001 | 8.10E-58 |
| SHBG | rs1881668 | 4 | 70725456 | G | C | 0.269 | 0.005 | 0.001 | 1.10E-08 |
| SHBG | rs28507491 | 4 | 77197651 | A | G | 0.377 | 0.013 | 0.001 | 1.20E-51 |
| SHBG | rs116243488 | 4 | 83245180 | T | G | 0.085 | 0.008 | 0.002 | 3.90E-08 |
| SHBG | rs13150068 | 4 | 88203828 | A | G | 0.563 | 0.018 | 0.001 | 1.40E-112 |
| SHBG | rs7678138 | 4 | 120106766 | G | A | 0.874 | 0.011 | 0.001 | 1.70E-18 |
| SHBG | rs1433210 | 4 | 124766956 | C | A | 0.246 | 0.008 | 0.001 | 2.90E-17 |
| SHBG | rs62334584 | 4 | 129031788 | T | C | 0.621 | 0.007 | 0.001 | 6.10E-16 |
| SHBG | rs28925904 | 4 | 144359490 | C | T | 0.975 | 0.021 | 0.003 | 3.60E-16 |
| SHBG | rs75686861 | 4 | 145621328 | G | A | 0.908 | 0.008 | 0.001 | 3.80E-10 |
| SHBG | rs10027275 | 4 | 148981496 | G | C | 0.259 | 0.011 | 0.001 | 5.70E-36 |
| SHBG | rs28367132 | 4 | 149639865 | A | G | 0.203 | 0.007 | 0.001 | 4.80E-11 |
| SHBG | rs72729610 | 4 | 154190965 | A | G | 0.833 | 0.010 | 0.001 | 8.10E-18 |
| SHBG | rs28730491 | 4 | 157681274 | C | G | 0.319 | 0.009 | 0.001 | 3.40E-24 |
| SHBG | rs78890745 | 4 | 159834474 | A | G | 0.109 | 0.017 | 0.001 | 1.70E-37 |
| SHBG | rs11729169 | 4 | 171019391 | T | C | 0.107 | 0.011 | 0.001 | 2.80E-15 |
| SHBG | rs11721999 | 4 | 185377222 | G | C | 0.165 | 0.007 | 0.001 | 9.90E-11 |
| SHBG | rs72709458 | 5 | 1283755 | C | T | 0.793 | 0.006 | 0.001 | 1.50E-09 |
| SHBG | rs4266430 | 5 | 38749736 | T | C | 0.488 | 0.005 | 0.001 | 1.50E-09 |
| SHBG | rs7735249 | 5 | 53310139 | C | G | 0.887 | 0.019 | 0.001 | 3.90E-46 |
| SHBG | rs40270 | 5 | 55804552 | A | C | 0.227 | 0.016 | 0.001 | 2.30E-60 |
| SHBG | rs79354983 | 5 | 56221537 | A | G | 0.905 | 0.012 | 0.001 | 5.20E-19 |
| SHBG | rs72753349 | 5 | 57392079 | T | C | 0.041 | 0.011 | 0.002 | 2.90E-08 |
| SHBG | rs4976033 | 5 | 67714246 | A | G | 0.599 | 0.007 | 0.001 | 9.70E-20 |
| SHBG | rs34651 | 5 | 72144005 | T | C | 0.919 | 0.012 | 0.002 | 8.60E-16 |
| SHBG | rs335629 | 5 | 76729521 | A | G | 0.485 | 0.004 | 0.001 | 1.10E-08 |
| SHBG | rs58729412 | 5 | 90169070 | C | T | 0.281 | 0.007 | 0.001 | 1.70E-13 |
| SHBG | rs13165542 | 5 | 122769296 | T | C | 0.824 | 0.008 | 0.001 | 6.70E-13 |
| SHBG | rs6860245 | 5 | 127367998 | C | G | 0.248 | 0.011 | 0.001 | 3.20E-30 |
| SHBG | rs2057655 | 5 | 131807624 | A | G | 0.186 | 0.010 | 0.001 | 2.80E-20 |
| SHBG | rs329120 | 5 | 133861756 | C | T | 0.581 | 0.009 | 0.001 | 2.30E-27 |
| SHBG | rs72802806 | 5 | 142798588 | G | A | 0.770 | 0.006 | 0.001 | 8.60E-09 |
| SHBG | rs2431752 | 5 | 162882702 | A | G | 0.107 | 0.010 | 0.001 | 3.50E-14 |
| SHBG | rs75049939 | 5 | 173334219 | T | G | 0.695 | 0.007 | 0.001 | 7.20E-18 |
| SHBG | rs1128287 | 5 | 176728458 | T | G | 0.777 | 0.005 | 0.001 | 1.80E-09 |
| SHBG | rs9379084 | 6 | 7231843 | G | A | 0.884 | 0.014 | 0.001 | 5.20E-26 |
| SHBG | rs2299055 | 6 | 15398331 | A | G | 0.118 | 0.007 | 0.001 | 1.00E-08 |
| SHBG | rs10946313 | 6 | 19381386 | T | C | 0.630 | 0.005 | 0.001 | 1.80E-11 |
| SHBG | rs4052755 | 6 | 19914496 | T | C | 0.292 | 0.006 | 0.001 | 1.40E-11 |
| SHBG | rs1408270 | 6 | 25873184 | G | A | 0.268 | 0.007 | 0.001 | 5.10E-16 |
| SHBG | rs9266184 | 6 | 31324664 | T | G | 0.681 | 0.011 | 0.001 | 3.30E-38 |
| SHBG | rs9461976 | 6 | 34237199 | G | A | 0.970 | 0.017 | 0.003 | 6.30E-10 |
| SHBG | rs4135240 | 6 | 36647680 | C | T | 0.330 | 0.007 | 0.001 | 3.00E-15 |
| SHBG | rs28360642 | 6 | 41667506 | A | C | 0.840 | 0.016 | 0.001 | 8.70E-48 |
| SHBG | rs1570360 | 6 | 43737830 | A | G | 0.329 | 0.006 | 0.001 | 2.70E-10 |
| SHBG | rs998584 | 6 | 43757896 | C | A | 0.518 | 0.009 | 0.001 | 2.10E-31 |
| SHBG | rs62407923 | 6 | 52391951 | T | C | 0.172 | 0.007 | 0.001 | 3.40E-09 |
| SHBG | rs12662365 | 6 | 80905389 | A | T | 0.321 | 0.005 | 0.001 | 8.00E-09 |
| SHBG | rs4472353 | 6 | 96078826 | C | T | 0.842 | 0.007 | 0.001 | 1.20E-08 |
| SHBG | rs17185536 | 6 | 100620931 | T | C | 0.244 | 0.007 | 0.001 | 1.70E-14 |
| SHBG | rs11153046 | 6 | 107441753 | A | G | 0.323 | 0.005 | 0.001 | 7.40E-10 |
| SHBG | rs9480889 | 6 | 109189021 | C | G | 0.217 | 0.006 | 0.001 | 2.10E-08 |
| SHBG | rs150115323 | 6 | 117506408 | G | C | 0.372 | 0.005 | 0.001 | 8.50E-12 |
| SHBG | rs6916491 | 6 | 119247734 | T | C | 0.707 | 0.005 | 0.001 | 2.10E-08 |
| SHBG | rs58321169 | 6 | 126868567 | C | T | 0.733 | 0.010 | 0.001 | 1.60E-25 |
| SHBG | rs12661232 | 6 | 130379160 | T | C | 0.312 | 0.011 | 0.001 | 7.70E-34 |
| SHBG | rs2908522 | 6 | 139835399 | G | C | 0.585 | 0.006 | 0.001 | 6.50E-12 |
| SHBG | rs11155787 | 6 | 151686905 | C | T | 0.360 | 0.006 | 0.001 | 1.10E-12 |
| SHBG | rs668871 | 6 | 160769811 | T | C | 0.469 | 0.018 | 0.001 | 1.40E-111 |
| SHBG | rs6919154 | 6 | 163743975 | A | G | 0.591 | 0.006 | 0.001 | 6.70E-12 |
| SHBG | rs4709746 | 6 | 164133001 | T | C | 0.134 | 0.010 | 0.001 | 6.40E-17 |
| SHBG | rs71538127 | 7 | 1010801 | C | G | 0.877 | 0.009 | 0.001 | 1.50E-13 |
| SHBG | rs62442919 | 7 | 1978384 | A | G | 0.382 | 0.008 | 0.001 | 1.50E-23 |
| SHBG | rs2462661 | 7 | 6702311 | G | T | 0.575 | 0.007 | 0.001 | 1.00E-16 |
| SHBG | rs38197 | 7 | 15909008 | C | G | 0.734 | 0.007 | 0.001 | 4.50E-13 |
| SHBG | rs2723572 | 7 | 17888587 | T | C | 0.488 | 0.005 | 0.001 | 3.20E-11 |
| SHBG | rs10951130 | 7 | 26385826 | T | G | 0.855 | 0.008 | 0.001 | 1.80E-12 |
| SHBG | rs10225221 | 7 | 35401635 | C | A | 0.299 | 0.005 | 0.001 | 3.10E-08 |
| SHBG | rs1799831 | 7 | 44199142 | C | T | 0.844 | 0.009 | 0.001 | 1.60E-13 |
| SHBG | rs7794048 | 7 | 46275882 | G | A | 0.937 | 0.013 | 0.002 | 4.80E-14 |
| SHBG | rs6975610 | 7 | 46642734 | G | A | 0.448 | 0.006 | 0.001 | 4.50E-11 |
| SHBG | rs12536766 | 7 | 70158864 | T | G | 0.570 | 0.006 | 0.001 | 4.10E-13 |
| SHBG | rs11770446 | 7 | 77358072 | G | A | 0.288 | 0.007 | 0.001 | 1.40E-19 |
| SHBG | rs1229492 | 7 | 81564122 | T | C | 0.268 | 0.011 | 0.001 | 1.30E-32 |
| SHBG | rs445 | 7 | 92408370 | C | T | 0.905 | 0.012 | 0.001 | 4.80E-18 |
| SHBG | rs7015 | 7 | 97920623 | G | A | 0.815 | 0.034 | 0.001 | 1.00E-200 |
| SHBG | rs1859690 | 7 | 99227172 | G | A | 0.067 | 0.013 | 0.002 | 7.10E-16 |
| SHBG | rs12667888 | 7 | 100474289 | C | T | 0.183 | 0.017 | 0.001 | 2.10E-59 |
| SHBG | rs149092986 | 7 | 111624089 | T | C | 0.976 | 0.016 | 0.003 | 2.40E-08 |
| SHBG | rs42374 | 7 | 116444070 | T | C | 0.547 | 0.006 | 0.001 | 8.60E-15 |
| SHBG | rs34748838 | 7 | 130459242 | T | C | 0.490 | 0.008 | 0.001 | 2.30E-20 |
| SHBG | rs157935 | 7 | 130585553 | G | T | 0.303 | 0.012 | 0.001 | 1.40E-39 |
| SHBG | rs200087953 | 7 | 133575017 | A | G | 0.682 | 0.005 | 0.001 | 2.60E-08 |
| SHBG | rs1559535 | 7 | 135143971 | G | A | 0.403 | 0.007 | 0.001 | 2.00E-17 |
| SHBG | rs34372369 | 7 | 143092269 | A | G | 0.052 | 0.016 | 0.002 | 2.00E-15 |
| SHBG | rs4725944 | 7 | 150476673 | C | G | 0.390 | 0.008 | 0.001 | 2.70E-21 |
| SHBG | rs114949263 | 7 | 150498245 | C | T | 0.111 | 0.015 | 0.001 | 1.70E-34 |
| SHBG | rs7808581 | 7 | 156194185 | C | T | 0.553 | 0.005 | 0.001 | 1.00E-09 |
| SHBG | rs4240624 | 8 | 9184231 | A | G | 0.908 | 0.022 | 0.001 | 2.30E-59 |
| SHBG | rs7464506 | 8 | 12624425 | A | T | 0.668 | 0.007 | 0.001 | 1.60E-13 |
| SHBG | rs113973451 | 8 | 21906789 | G | T | 0.823 | 0.007 | 0.001 | 7.70E-13 |
| SHBG | rs2241261 | 8 | 22876739 | C | T | 0.476 | 0.006 | 0.001 | 2.40E-14 |
| SHBG | rs9644032 | 8 | 23414822 | T | G | 0.367 | 0.007 | 0.001 | 7.30E-18 |
| SHBG | rs4739515 | 8 | 37391203 | C | G | 0.041 | 0.012 | 0.002 | 1.90E-08 |
| SHBG | rs117318607 | 8 | 38947274 | G | A | 0.022 | 0.015 | 0.003 | 2.50E-09 |
| SHBG | rs12543287 | 8 | 42334511 | C | G | 0.371 | 0.010 | 0.001 | 2.40E-37 |
| SHBG | rs72656017 | 8 | 57152481 | A | G | 0.870 | 0.008 | 0.001 | 6.40E-12 |
| SHBG | rs10504255 | 8 | 59398461 | A | G | 0.662 | 0.011 | 0.001 | 3.00E-36 |
| SHBG | rs72663955 | 8 | 71139330 | T | G | 0.867 | 0.007 | 0.001 | 5.30E-09 |
| SHBG | rs113605295 | 8 | 81374095 | T | C | 0.064 | 0.012 | 0.002 | 3.40E-14 |
| SHBG | rs76767219 | 8 | 81426196 | A | C | 0.035 | 0.043 | 0.002 | 6.90E-83 |
| SHBG | rs7828742 | 8 | 116960729 | A | G | 0.401 | 0.008 | 0.001 | 1.10E-20 |
| SHBG | rs11774700 | 8 | 118220270 | C | T | 0.309 | 0.006 | 0.001 | 7.30E-13 |
| SHBG | rs4871015 | 8 | 128314516 | A | G | 0.581 | 0.004 | 0.001 | 5.00E-08 |
| SHBG | rs55831924 | 8 | 145031968 | T | C | 0.361 | 0.007 | 0.001 | 3.50E-15 |
| SHBG | rs2721195 | 8 | 145677011 | T | C | 0.474 | 0.010 | 0.001 | 4.20E-33 |
| SHBG | rs1567353 | 9 | 1033773 | C | G | 0.692 | 0.008 | 0.001 | 6.60E-18 |
| SHBG | rs10757112 | 9 | 2011588 | A | G | 0.603 | 0.005 | 0.001 | 2.10E-08 |
| SHBG | rs10114763 | 9 | 4143749 | T | A | 0.423 | 0.009 | 0.001 | 3.80E-26 |
| SHBG | rs1330307 | 9 | 4305064 | A | C | 0.514 | 0.007 | 0.001 | 7.90E-18 |
| SHBG | rs79793188 | 9 | 6449701 | C | T | 0.812 | 0.006 | 0.001 | 1.00E-08 |
| SHBG | rs820504 | 9 | 6668278 | G | A | 0.864 | 0.012 | 0.001 | 1.70E-24 |
| SHBG | rs2031316 | 9 | 13563624 | A | C | 0.081 | 0.010 | 0.002 | 4.50E-10 |
| SHBG | rs10811662 | 9 | 22134253 | A | G | 0.173 | 0.007 | 0.001 | 1.10E-10 |
| SHBG | rs35234337 | 9 | 35661243 | C | T | 0.742 | 0.006 | 0.001 | 3.30E-08 |
| SHBG | rs10868080 | 9 | 86626769 | T | A | 0.256 | 0.023 | 0.001 | 3.40E-125 |
| SHBG | rs143554698 | 9 | 95538573 | C | T | 0.859 | 0.008 | 0.001 | 1.50E-12 |
| SHBG | rs1475543 | 9 | 100333671 | C | A | 0.827 | 0.006 | 0.001 | 1.40E-08 |
| SHBG | rs11515536 | 9 | 101771183 | T | C | 0.131 | 0.007 | 0.001 | 1.60E-08 |
| SHBG | rs4743776 | 9 | 107727727 | G | A | 0.299 | 0.006 | 0.001 | 1.30E-10 |
| SHBG | rs62580766 | 9 | 113034490 | T | C | 0.182 | 0.011 | 0.001 | 3.20E-27 |
| SHBG | rs6477768 | 9 | 113168239 | A | G | 0.682 | 0.006 | 0.001 | 2.50E-09 |
| SHBG | rs17372936 | 9 | 119066203 | T | C | 0.768 | 0.008 | 0.001 | 4.10E-18 |
| SHBG | rs4837794 | 9 | 123507855 | T | C | 0.331 | 0.008 | 0.001 | 1.70E-22 |
| SHBG | rs700085 | 9 | 125870466 | T | C | 0.088 | 0.009 | 0.001 | 3.70E-11 |
| SHBG | rs9697210 | 9 | 131468740 | G | A | 0.854 | 0.016 | 0.001 | 9.20E-45 |
| SHBG | rs8176693 | 9 | 136137657 | C | T | 0.938 | 0.013 | 0.002 | 7.40E-14 |
| SHBG | rs72766607 | 9 | 136895818 | T | G | 0.980 | 0.034 | 0.003 | 5.20E-32 |
| SHBG | rs11791747 | 9 | 137106879 | G | A | 0.307 | 0.007 | 0.001 | 6.90E-15 |
| SHBG | rs35233014 | 9 | 137268177 | C | A | 0.254 | 0.014 | 0.001 | 2.50E-46 |
| SHBG | rs11103377 | 9 | 139097135 | G | A | 0.539 | 0.010 | 0.001 | 1.20E-32 |
| SHBG | rs2279415 | 10 | 3792608 | G | A | 0.531 | 0.005 | 0.001 | 1.90E-08 |
| SHBG | rs79717793 | 10 | 5262267 | G | A | 0.845 | 0.021 | 0.001 | 8.50E-84 |
| SHBG | rs3824655 | 10 | 13370779 | C | G | 0.590 | 0.006 | 0.001 | 7.30E-14 |
| SHBG | rs3737178 | 10 | 31607215 | A | G | 0.951 | 0.010 | 0.002 | 1.20E-08 |
| SHBG | rs899865 | 10 | 36473044 | T | C | 0.600 | 0.005 | 0.001 | 1.20E-08 |
| SHBG | rs72783094 | 10 | 49686734 | A | G | 0.908 | 0.008 | 0.001 | 1.80E-08 |
| SHBG | rs1530439 | 10 | 63645959 | T | G | 0.309 | 0.009 | 0.001 | 7.90E-24 |
| SHBG | rs541030121 | 10 | 64871859 | A | G | 0.065 | 0.016 | 0.002 | 5.50E-15 |
| SHBG | rs537858427 | 10 | 65097399 | G | A | 0.100 | 0.010 | 0.002 | 1.50E-08 |
| SHBG | rs10822163 | 10 | 65124098 | G | C | 0.473 | 0.053 | 0.001 | 1.00E-200 |
| SHBG | rs1204083 | 10 | 69835197 | C | G | 0.346 | 0.007 | 0.001 | 2.50E-17 |
| SHBG | rs7100001 | 10 | 74654754 | A | G | 0.049 | 0.011 | 0.002 | 1.30E-08 |
| SHBG | rs2579162 | 10 | 79541639 | C | T | 0.709 | 0.004 | 0.001 | 1.50E-08 |
| SHBG | rs1782652 | 10 | 81074125 | T | A | 0.619 | 0.012 | 0.001 | 2.90E-50 |
| SHBG | rs11202594 | 10 | 89641222 | G | A | 0.843 | 0.006 | 0.001 | 9.40E-09 |
| SHBG | rs1772189 | 10 | 93629499 | T | A | 0.523 | 0.012 | 0.001 | 9.40E-54 |
| SHBG | rs2068888 | 10 | 94839642 | A | G | 0.450 | 0.011 | 0.001 | 1.70E-40 |
| SHBG | rs7080472 | 10 | 96012950 | T | G | 0.423 | 0.005 | 0.001 | 1.30E-08 |
| SHBG | rs11188601 | 10 | 97856899 | C | T | 0.364 | 0.008 | 0.001 | 1.20E-22 |
| SHBG | rs2862954 | 10 | 101912064 | C | T | 0.500 | 0.010 | 0.001 | 3.30E-28 |
| SHBG | rs67477288 | 10 | 103587858 | C | T | 0.942 | 0.010 | 0.002 | 2.70E-08 |
| SHBG | rs80235628 | 10 | 122859270 | G | A | 0.950 | 0.018 | 0.002 | 2.30E-21 |
| SHBG | rs7893136 | 10 | 122926460 | T | C | 0.021 | 0.018 | 0.003 | 5.50E-10 |
| SHBG | rs4758639 | 11 | 305406 | A | G | 0.664 | 0.007 | 0.001 | 1.00E-13 |
| SHBG | rs2412138 | 11 | 3090126 | T | C | 0.606 | 0.006 | 0.001 | 5.30E-13 |
| SHBG | rs11601507 | 11 | 5701074 | A | C | 0.069 | 0.016 | 0.002 | 1.10E-25 |
| SHBG | rs1037169 | 11 | 13361005 | T | C | 0.313 | 0.012 | 0.001 | 1.20E-41 |
| SHBG | rs2074310 | 11 | 17421886 | C | T | 0.643 | 0.006 | 0.001 | 1.50E-12 |
| SHBG | rs62618693 | 11 | 32956492 | T | C | 0.045 | 0.016 | 0.002 | 2.30E-16 |
| SHBG | rs4756190 | 11 | 35124040 | T | C | 0.580 | 0.004 | 0.001 | 1.00E-08 |
| SHBG | rs2292910 | 11 | 45903613 | A | C | 0.332 | 0.006 | 0.001 | 1.20E-11 |
| SHBG | rs566217606 | 11 | 48877002 | C | T | 0.013 | 0.022 | 0.004 | 1.40E-08 |
| SHBG | rs143709973 | 11 | 59572669 | A | C | 0.955 | 0.012 | 0.002 | 4.40E-10 |
| SHBG | rs174533 | 11 | 61549025 | G | A | 0.652 | 0.010 | 0.001 | 1.20E-28 |
| SHBG | rs12797706 | 11 | 65561369 | A | G | 0.235 | 0.014 | 0.001 | 3.20E-41 |
| SHBG | rs4988308 | 11 | 68106842 | G | A | 0.557 | 0.005 | 0.001 | 1.20E-09 |
| SHBG | rs631695 | 11 | 69283303 | T | G | 0.416 | 0.014 | 0.001 | 6.80E-68 |
| SHBG | rs75713100 | 11 | 77517973 | T | G | 0.911 | 0.008 | 0.001 | 3.50E-09 |
| SHBG | rs12575636 | 11 | 95311260 | T | G | 0.811 | 0.010 | 0.001 | 2.60E-18 |
| SHBG | rs10895276 | 11 | 102083695 | C | T | 0.658 | 0.009 | 0.001 | 3.80E-25 |
| SHBG | rs78312641 | 11 | 118749318 | A | T | 0.128 | 0.008 | 0.001 | 2.50E-10 |
| SHBG | rs55771168 | 11 | 119070949 | C | T | 0.272 | 0.007 | 0.001 | 5.70E-15 |
| SHBG | rs2156804 | 11 | 122610326 | G | T | 0.513 | 0.005 | 0.001 | 1.20E-09 |
| SHBG | rs2369280 | 12 | 508859 | T | G | 0.737 | 0.005 | 0.001 | 3.00E-08 |
| SHBG | rs56196860 | 12 | 2908330 | A | C | 0.031 | 0.023 | 0.002 | 4.40E-24 |
| SHBG | rs76895963 | 12 | 4384844 | G | T | 0.021 | 0.073 | 0.003 | 1.20E-120 |
| SHBG | rs3782735 | 12 | 6885076 | A | G | 0.600 | 0.008 | 0.001 | 1.20E-21 |
| SHBG | rs11045171 | 12 | 20470199 | G | A | 0.198 | 0.008 | 0.001 | 1.30E-14 |
| SHBG | rs57743625 | 12 | 21367633 | G | A | 0.840 | 0.030 | 0.001 | 1.10E-159 |
| SHBG | rs2900528 | 12 | 24150441 | C | G | 0.417 | 0.006 | 0.001 | 1.50E-12 |
| SHBG | rs1391790 | 12 | 24203096 | T | C | 0.964 | 0.025 | 0.002 | 1.80E-30 |
| SHBG | rs75130744 | 12 | 25410741 | G | C | 0.929 | 0.027 | 0.002 | 7.30E-72 |
| SHBG | rs2129869 | 12 | 26457650 | A | T | 0.780 | 0.007 | 0.001 | 1.20E-12 |
| SHBG | rs34072608 | 12 | 46189284 | G | T | 0.801 | 0.007 | 0.001 | 1.60E-13 |
| SHBG | rs118080406 | 12 | 47249428 | G | A | 0.025 | 0.015 | 0.003 | 2.30E-08 |
| SHBG | rs56365029 | 12 | 49036347 | A | G | 0.978 | 0.017 | 0.003 | 1.00E-10 |
| SHBG | rs864899 | 12 | 51221127 | A | G | 0.415 | 0.012 | 0.001 | 5.20E-47 |
| SHBG | rs12818938 | 12 | 53783182 | T | G | 0.832 | 0.009 | 0.001 | 4.10E-14 |
| SHBG | rs73139029 | 12 | 62822406 | A | T | 0.859 | 0.007 | 0.001 | 2.40E-09 |
| SHBG | rs145775785 | 12 | 65902265 | T | C | 0.015 | 0.023 | 0.004 | 6.10E-10 |
| SHBG | rs2583939 | 12 | 66213521 | C | T | 0.868 | 0.008 | 0.001 | 6.40E-10 |
| SHBG | rs1042725 | 12 | 66358347 | C | T | 0.508 | 0.008 | 0.001 | 7.50E-25 |
| SHBG | rs11176664 | 12 | 67661519 | A | G | 0.495 | 0.005 | 0.001 | 3.40E-09 |
| SHBG | rs2601007 | 12 | 69979115 | G | C | 0.654 | 0.005 | 0.001 | 2.70E-10 |
| SHBG | rs374335 | 12 | 77457439 | A | G | 0.681 | 0.006 | 0.001 | 2.80E-10 |
| SHBG | rs11107124 | 12 | 93988283 | G | C | 0.711 | 0.006 | 0.001 | 2.50E-10 |
| SHBG | rs11108061 | 12 | 95857620 | T | C | 0.427 | 0.005 | 0.001 | 8.70E-09 |
| SHBG | rs11111274 | 12 | 102838128 | G | A | 0.263 | 0.010 | 0.001 | 2.50E-25 |
| SHBG | rs112725417 | 12 | 112657361 | T | C | 0.973 | 0.017 | 0.003 | 3.50E-11 |
| SHBG | rs4767327 | 12 | 115929191 | T | A | 0.618 | 0.005 | 0.001 | 7.90E-10 |
| SHBG | rs113257350 | 12 | 120381998 | C | T | 0.978 | 0.017 | 0.003 | 4.60E-10 |
| SHBG | rs2393775 | 12 | 121424574 | A | G | 0.622 | 0.015 | 0.001 | 2.00E-80 |
| SHBG | rs73214164 | 12 | 121470978 | T | C | 0.802 | 0.010 | 0.001 | 4.90E-22 |
| SHBG | rs12311848 | 12 | 124486851 | G | A | 0.334 | 0.010 | 0.001 | 1.10E-33 |
| SHBG | rs1725788 | 12 | 131608476 | G | A | 0.238 | 0.006 | 0.001 | 3.30E-09 |
| SHBG | rs749170 | 13 | 22350875 | T | C | 0.335 | 0.005 | 0.001 | 7.00E-10 |
| SHBG | rs9533843 | 13 | 44980150 | A | G | 0.466 | 0.005 | 0.001 | 1.80E-08 |
| SHBG | rs41284816 | 13 | 50655989 | T | G | 0.019 | 0.031 | 0.003 | 1.50E-25 |
| SHBG | rs3116625 | 13 | 50945251 | C | T | 0.800 | 0.007 | 0.001 | 1.10E-13 |
| SHBG | rs11843816 | 13 | 91987065 | C | T | 0.965 | 0.013 | 0.002 | 3.00E-08 |
| SHBG | rs750598 | 13 | 111028978 | A | G | 0.338 | 0.005 | 0.001 | 9.50E-09 |
| SHBG | rs373373 | 13 | 111297952 | A | C | 0.948 | 0.011 | 0.002 | 1.30E-09 |
| SHBG | rs116338429 | 13 | 114767040 | T | C | 0.171 | 0.008 | 0.001 | 2.10E-11 |
| SHBG | rs112035922 | 13 | 115047464 | C | T | 0.768 | 0.008 | 0.001 | 1.90E-18 |
| SHBG | rs2064482 | 14 | 23709315 | T | C | 0.255 | 0.012 | 0.001 | 1.50E-43 |
| SHBG | rs11621792 | 14 | 24871926 | C | T | 0.547 | 0.019 | 0.001 | 1.80E-117 |
| SHBG | rs12435790 | 14 | 35154381 | G | A | 0.078 | 0.010 | 0.002 | 5.80E-10 |
| SHBG | rs2239222 | 14 | 73011885 | G | A | 0.349 | 0.011 | 0.001 | 9.40E-35 |
| SHBG | rs13379043 | 14 | 74250126 | C | T | 0.279 | 0.010 | 0.001 | 2.60E-28 |
| SHBG | rs1005421 | 14 | 89886940 | C | T | 0.584 | 0.007 | 0.001 | 7.60E-20 |
| SHBG | rs28929474 | 14 | 94844947 | T | C | 0.020 | 0.096 | 0.003 | 1.00E-200 |
| SHBG | rs17580 | 14 | 94847262 | A | T | 0.048 | 0.026 | 0.002 | 1.90E-42 |
| SHBG | rs3742366 | 14 | 104198351 | C | T | 0.346 | 0.008 | 0.001 | 9.70E-23 |
| SHBG | rs2498786 | 14 | 105262368 | C | G | 0.384 | 0.010 | 0.001 | 1.50E-32 |
| SHBG | rs56112295 | 14 | 105877057 | T | C | 0.226 | 0.006 | 0.001 | 1.60E-10 |
| SHBG | rs28510484 | 15 | 31637569 | G | C | 0.829 | 0.008 | 0.001 | 3.80E-12 |
| SHBG | rs55800572 | 15 | 35285183 | G | C | 0.339 | 0.008 | 0.001 | 6.80E-20 |
| SHBG | rs2454352 | 15 | 36047091 | C | T | 0.239 | 0.007 | 0.001 | 1.80E-12 |
| SHBG | rs275177 | 15 | 39449003 | C | T | 0.149 | 0.008 | 0.001 | 2.70E-11 |
| SHBG | rs28790585 | 15 | 39668870 | C | T | 0.705 | 0.006 | 0.001 | 1.50E-09 |
| SHBG | rs11637681 | 15 | 40387971 | A | G | 0.723 | 0.008 | 0.001 | 2.20E-18 |
| SHBG | rs139974673 | 15 | 44027885 | T | C | 0.975 | 0.066 | 0.003 | 5.10E-147 |
| SHBG | rs1008805 | 15 | 51549599 | A | G | 0.576 | 0.005 | 0.001 | 6.10E-09 |
| SHBG | rs181598957 | 15 | 53327792 | A | G | 0.018 | 0.024 | 0.003 | 3.50E-13 |
| SHBG | rs79391862 | 15 | 53739426 | A | C | 0.986 | 0.082 | 0.004 | 2.20E-121 |
| SHBG | rs4775191 | 15 | 59926946 | A | C | 0.339 | 0.007 | 0.001 | 9.80E-16 |
| SHBG | rs339998 | 15 | 60947763 | T | C | 0.394 | 0.005 | 0.001 | 5.10E-09 |
| SHBG | rs3848125 | 15 | 61957236 | A | G | 0.577 | 0.005 | 0.001 | 1.50E-11 |
| SHBG | rs76428668 | 15 | 66839282 | T | G | 0.750 | 0.009 | 0.001 | 9.10E-24 |
| SHBG | rs8038465 | 15 | 73978337 | T | C | 0.425 | 0.005 | 0.001 | 3.00E-10 |
| SHBG | rs112500920 | 15 | 82507605 | T | C | 0.930 | 0.012 | 0.002 | 4.00E-11 |
| SHBG | rs9672839 | 15 | 93568884 | C | A | 0.395 | 0.005 | 0.001 | 6.50E-09 |
| SHBG | rs12898856 | 15 | 96227920 | C | T | 0.551 | 0.010 | 0.001 | 5.40E-36 |
| SHBG | rs56332871 | 15 | 96714816 | A | C | 0.272 | 0.035 | 0.001 | 1.00E-200 |
| SHBG | rs28469124 | 16 | 1842973 | C | G | 0.087 | 0.010 | 0.001 | 6.60E-13 |
| SHBG | rs28372698 | 16 | 3115111 | A | T | 0.406 | 0.005 | 0.001 | 6.00E-09 |
| SHBG | rs3747587 | 16 | 4674954 | G | C | 0.814 | 0.010 | 0.001 | 3.00E-21 |
| SHBG | rs720130 | 16 | 11132633 | G | T | 0.595 | 0.005 | 0.001 | 5.10E-12 |
| SHBG | rs3743588 | 16 | 11836508 | G | A | 0.715 | 0.007 | 0.001 | 9.00E-17 |
| SHBG | rs12928099 | 16 | 15150505 | A | C | 0.295 | 0.010 | 0.001 | 4.00E-29 |
| SHBG | rs41278174 | 16 | 16259596 | A | G | 0.027 | 0.013 | 0.003 | 3.00E-08 |
| SHBG | rs34050011 | 16 | 49868513 | A | C | 0.424 | 0.006 | 0.001 | 1.20E-09 |
| SHBG | rs11643656 | 16 | 51463539 | G | A | 0.825 | 0.007 | 0.001 | 5.40E-09 |
| SHBG | rs246192 | 16 | 58544295 | G | C | 0.479 | 0.007 | 0.001 | 1.40E-16 |
| SHBG | rs61733486 | 16 | 68390697 | C | T | 0.942 | 0.011 | 0.002 | 5.60E-10 |
| SHBG | rs77147683 | 16 | 71623594 | A | C | 0.233 | 0.005 | 0.001 | 8.70E-09 |
| SHBG | rs2925979 | 16 | 81534790 | C | T | 0.699 | 0.007 | 0.001 | 2.30E-16 |
| SHBG | rs4782568 | 16 | 83980529 | G | C | 0.452 | 0.013 | 0.001 | 4.30E-58 |
| SHBG | rs11641834 | 16 | 88070573 | C | T | 0.569 | 0.010 | 0.001 | 1.00E-36 |
| SHBG | rs56292801 | 16 | 88535341 | A | G | 0.273 | 0.009 | 0.001 | 1.00E-25 |
| SHBG | rs11078597 | 17 | 1618363 | C | T | 0.186 | 0.017 | 0.001 | 2.70E-61 |
| SHBG | rs9902384 | 17 | 5171746 | A | G | 0.753 | 0.006 | 0.001 | 8.20E-09 |
| SHBG | rs858519 | 17 | 7531965 | C | T | 0.557 | 0.097 | 0.001 | 1.00E-200 |
| SHBG | rs8066941 | 17 | 9588450 | T | G | 0.762 | 0.012 | 0.001 | 5.90E-40 |
| SHBG | rs17669311 | 17 | 13837051 | G | A | 0.611 | 0.009 | 0.001 | 2.10E-29 |
| SHBG | rs12937088 | 17 | 16031029 | G | A | 0.508 | 0.006 | 0.001 | 8.50E-17 |
| SHBG | rs8079418 | 17 | 17924060 | C | T | 0.612 | 0.014 | 0.001 | 3.70E-65 |
| SHBG | rs12943365 | 17 | 29680526 | G | C | 0.609 | 0.009 | 0.001 | 5.50E-30 |
| SHBG | rs17138478 | 17 | 36073320 | A | C | 0.129 | 0.009 | 0.001 | 3.30E-14 |
| SHBG | rs17616365 | 17 | 38256401 | G | A | 0.968 | 0.022 | 0.002 | 3.20E-23 |
| SHBG | rs650558 | 17 | 40721042 | C | T | 0.751 | 0.007 | 0.001 | 2.20E-14 |
| SHBG | rs10048173 | 17 | 45592266 | G | A | 0.513 | 0.015 | 0.001 | 1.50E-80 |
| SHBG | rs11655704 | 17 | 47448172 | C | T | 0.314 | 0.032 | 0.001 | 1.00E-200 |
| SHBG | rs8077316 | 17 | 48626389 | T | C | 0.246 | 0.006 | 0.001 | 1.60E-10 |
| SHBG | rs8074363 | 17 | 59241469 | C | T | 0.775 | 0.006 | 0.001 | 6.10E-11 |
| SHBG | rs76708468 | 17 | 62206299 | T | C | 0.961 | 0.013 | 0.002 | 4.50E-09 |
| SHBG | rs17650301 | 17 | 62479273 | C | A | 0.294 | 0.005 | 0.001 | 1.20E-09 |
| SHBG | rs1801689 | 17 | 64210580 | A | C | 0.970 | 0.038 | 0.002 | 3.00E-60 |
| SHBG | rs7211695 | 17 | 65235971 | A | G | 0.521 | 0.008 | 0.001 | 1.40E-24 |
| SHBG | rs34931250 | 17 | 66879927 | T | C | 0.061 | 0.010 | 0.002 | 6.10E-10 |
| SHBG | rs9282552 | 17 | 67109894 | T | C | 0.650 | 0.007 | 0.001 | 4.70E-18 |
| SHBG | rs1605750 | 17 | 68471073 | A | G | 0.510 | 0.005 | 0.001 | 4.60E-10 |
| SHBG | rs72844546 | 17 | 73149850 | C | T | 0.346 | 0.010 | 0.001 | 2.80E-33 |
| SHBG | rs2587505 | 17 | 77784268 | T | C | 0.580 | 0.006 | 0.001 | 1.30E-11 |
| SHBG | rs10153315 | 17 | 79481772 | T | C | 0.582 | 0.008 | 0.001 | 2.90E-24 |
| SHBG | rs11664106 | 18 | 2846812 | T | A | 0.373 | 0.007 | 0.001 | 7.10E-17 |
| SHBG | rs9945126 | 18 | 46469962 | C | G | 0.465 | 0.004 | 0.001 | 1.30E-08 |
| SHBG | rs55855238 | 18 | 55089715 | C | T | 0.650 | 0.010 | 0.001 | 5.10E-29 |
| SHBG | rs6567230 | 18 | 59330824 | T | A | 0.701 | 0.006 | 0.001 | 1.60E-11 |
| SHBG | rs620068 | 18 | 60140576 | A | G | 0.459 | 0.005 | 0.001 | 7.60E-10 |
| SHBG | rs12454712 | 18 | 60845884 | C | T | 0.377 | 0.010 | 0.001 | 1.00E-32 |
| SHBG | rs3829639 | 18 | 71943144 | A | G | 0.672 | 0.008 | 0.001 | 1.30E-19 |
| SHBG | rs34668346 | 19 | 672169 | A | G | 0.513 | 0.005 | 0.001 | 8.90E-10 |
| SHBG | rs150122016 | 19 | 1246079 | G | T | 0.972 | 0.016 | 0.003 | 8.10E-09 |
| SHBG | rs1640269 | 19 | 2793194 | C | A | 0.286 | 0.019 | 0.001 | 9.10E-101 |
| SHBG | rs11539938 | 19 | 3062857 | C | T | 0.421 | 0.010 | 0.001 | 2.40E-29 |
| SHBG | rs60018147 | 19 | 3375572 | G | A | 0.120 | 0.014 | 0.001 | 2.60E-25 |
| SHBG | rs10221473 | 19 | 7236626 | G | A | 0.542 | 0.007 | 0.001 | 6.60E-13 |
| SHBG | rs8107967 | 19 | 7972615 | G | A | 0.567 | 0.007 | 0.001 | 1.30E-15 |
| SHBG | rs281439 | 19 | 10400110 | C | G | 0.780 | 0.006 | 0.001 | 3.00E-08 |
| SHBG | rs8101895 | 19 | 12508061 | A | T | 0.236 | 0.007 | 0.001 | 3.50E-13 |
| SHBG | rs7252372 | 19 | 14172896 | G | C | 0.556 | 0.009 | 0.001 | 8.20E-27 |
| SHBG | rs202200760 | 19 | 17346854 | C | G | 0.039 | 0.073 | 0.002 | 1.00E-200 |
| SHBG | rs4805881 | 19 | 33896432 | C | A | 0.666 | 0.009 | 0.001 | 4.30E-29 |
| SHBG | rs45512696 | 19 | 35550878 | T | C | 0.175 | 0.021 | 0.001 | 2.00E-86 |
| SHBG | rs11666245 | 19 | 38229926 | G | A | 0.953 | 0.017 | 0.002 | 7.00E-20 |
| SHBG | rs11673023 | 19 | 38239521 | C | A | 0.829 | 0.007 | 0.001 | 9.60E-12 |
| SHBG | rs483082 | 19 | 45416178 | T | G | 0.237 | 0.009 | 0.001 | 1.90E-21 |
| SHBG | rs5112 | 19 | 45430280 | G | C | 0.533 | 0.008 | 0.001 | 4.90E-20 |
| SHBG | rs34255979 | 19 | 46384830 | T | C | 0.120 | 0.028 | 0.001 | 1.50E-108 |
| SHBG | rs111981233 | 19 | 50016479 | G | T | 0.080 | 0.021 | 0.002 | 1.60E-46 |
| SHBG | rs11672485 | 19 | 53833712 | C | T | 0.490 | 0.005 | 0.001 | 1.10E-12 |
| SHBG | rs4077285 | 19 | 56599405 | G | C | 0.906 | 0.012 | 0.001 | 1.90E-15 |
| SHBG | rs16988208 | 19 | 58332315 | G | T | 0.164 | 0.007 | 0.001 | 1.70E-10 |
| SHBG | rs11545185 | 19 | 59028585 | A | G | 0.171 | 0.009 | 0.001 | 2.20E-16 |
| SHBG | rs144033177 | 20 | 571467 | A | C | 0.984 | 0.018 | 0.003 | 1.10E-08 |
| SHBG | rs1741288 | 20 | 4102954 | A | G | 0.635 | 0.005 | 0.001 | 2.30E-11 |
| SHBG | rs7261425 | 20 | 20068635 | G | C | 0.278 | 0.005 | 0.001 | 2.70E-08 |
| SHBG | rs6048205 | 20 | 22559601 | A | G | 0.957 | 0.012 | 0.002 | 3.60E-08 |
| SHBG | rs13042148 | 20 | 32298286 | C | T | 0.845 | 0.014 | 0.001 | 9.00E-39 |
| SHBG | rs6029640 | 20 | 39970385 | G | A | 0.420 | 0.010 | 0.001 | 9.60E-33 |
| SHBG | rs6073431 | 20 | 43040569 | T | C | 0.531 | 0.017 | 0.001 | 5.10E-92 |
| SHBG | rs1412957 | 20 | 45557065 | A | G | 0.574 | 0.007 | 0.001 | 3.50E-16 |
| SHBG | rs6018424 | 20 | 45986984 | T | C | 0.199 | 0.008 | 0.001 | 4.40E-14 |
| SHBG | rs55987409 | 20 | 49569025 | T | C | 0.072 | 0.018 | 0.002 | 6.40E-28 |
| SHBG | rs61744628 | 20 | 52186837 | A | G | 0.027 | 0.017 | 0.003 | 1.70E-11 |
| SHBG | rs6123685 | 20 | 55836040 | A | G | 0.254 | 0.005 | 0.001 | 3.80E-09 |
| SHBG | rs62217799 | 20 | 62347191 | G | T | 0.342 | 0.007 | 0.001 | 2.20E-15 |
| SHBG | rs1475883 | 21 | 17591913 | G | A | 0.567 | 0.004 | 0.001 | 2.40E-08 |
| SHBG | rs112078975 | 21 | 33095959 | G | A | 0.951 | 0.012 | 0.002 | 8.40E-11 |
| SHBG | rs4818008 | 21 | 40611442 | T | A | 0.645 | 0.005 | 0.001 | 2.70E-08 |
| SHBG | rs35598889 | 22 | 18450794 | C | T | 0.233 | 0.007 | 0.001 | 8.50E-15 |
| SHBG | rs759404 | 22 | 18916180 | C | T | 0.932 | 0.010 | 0.002 | 4.00E-08 |
| SHBG | rs9606233 | 22 | 20066611 | C | G | 0.405 | 0.005 | 0.001 | 8.90E-12 |
| SHBG | rs4820091 | 22 | 21940189 | G | T | 0.181 | 0.011 | 0.001 | 1.60E-21 |
| SHBG | rs6005840 | 22 | 29101357 | A | G | 0.326 | 0.012 | 0.001 | 3.00E-42 |
| SHBG | rs5749082 | 22 | 30770603 | T | A | 0.292 | 0.011 | 0.001 | 3.10E-36 |
| SHBG | rs9610329 | 22 | 36042986 | C | T | 0.572 | 0.006 | 0.001 | 7.60E-13 |
| SHBG | rs13057133 | 22 | 38179473 | C | T | 0.699 | 0.007 | 0.001 | 3.00E-15 |
| SHBG | rs2075915 | 22 | 39254556 | A | G | 0.747 | 0.006 | 0.001 | 2.40E-11 |
| SHBG | rs738409 | 22 | 44324727 | G | C | 0.216 | 0.023 | 0.001 | 3.10E-114 |
| SHBG | rs135563 | 22 | 46536017 | T | C | 0.510 | 0.005 | 0.001 | 1.70E-09 |
| SHBG | rs36171610 | 22 | 50422385 | G | A | 0.509 | 0.006 | 0.001 | 2.00E-11 |
| Estradiol | rs1260326 | 2 | 27730940 | C | T | 0.606 | 0.006 | 0.001 | 9.60E-11 |
| Estradiol | rs112881196 | 2 | 31982811 | G | C | 0.039 | 0.025 | 0.002 | 5.60E-30 |
| Estradiol | rs45446698 | 7 | 99332948 | T | G | 0.958 | 0.016 | 0.002 | 7.90E-14 |
| Estradiol | rs657152 | 9 | 136139265 | C | A | 0.661 | 0.008 | 0.001 | 5.50E-21 |
| Estradiol | rs56196860 | 12 | 2908330 | A | C | 0.031 | 0.021 | 0.002 | 2.60E-18 |
| Estradiol | rs34019140 | 14 | 106527500 | G | A | 0.564 | 0.012 | 0.001 | 6.90E-42 |
| Estradiol | rs7173595 | 15 | 51533736 | T | C | 0.649 | 0.016 | 0.001 | 3.60E-72 |
| Estradiol | rs3751591 | 15 | 51606710 | G | A | 0.167 | 0.008 | 0.001 | 6.60E-12 |
| Estradiol | rs727428 | 17 | 7537792 | C | T | 0.557 | 0.006 | 0.001 | 1.80E-11 |
| Estradiol | rs113047993 | 18 | 20585399 | C | T | 0.931 | 0.010 | 0.002 | 8.70E-09 |
| Estradiol | rs201687269 | 18 | 27520799 | T | A | 0.675 | 0.006 | 0.001 | 1.80E-08 |
| Estradiol | rs10425629 | 19 | 48384648 | C | T | 0.165 | 0.008 | 0.001 | 2.70E-11 |
| Estradiol | rs117826558 | 22 | 46770756 | T | C | 0.034 | 0.014 | 0.002 | 2.70E-08 |
| IGF-1 | rs7535144 | 1 | 154965113 | G | T | 0.029 | -0.040 | 0.007 | 1.44E-09 |
| IGF-1 | rs121912698 | 3 | 52022837 | C | T | 0.004 | 0.163 | 0.019 | 6.42E-18 |
| IGF-1 | rs1229984 | 4 | 100239319 | T | C | 0.022 | -0.092 | 0.008 | 3.31E-32 |
| IGF-1 | rs17714046 | 5 | 180661980 | T | C | 0.046 | 0.041 | 0.006 | 5.38E-13 |
| IGF-1 | rs913455 | 6 | 25420344 | C | G | 0.089 | 0.029 | 0.004 | 1.66E-11 |
| IGF-1 | rs71844696 | 7 | 6714788 | CCTT | C | 0.097 | 0.026 | 0.004 | 1.17E-10 |
| IGF-1 | rs199761265 | 7 | 6731803 | G | A | 0.002 | 0.192 | 0.030 | 1.80E-10 |
| IGF-1 | rs2228078 | 7 | 31018852 | T | C | 0.015 | 0.058 | 0.010 | 1.58E-09 |
| IGF-1 | rs9282734 | 7 | 45956969 | T | G | 0.004 | 0.177 | 0.018 | 1.59E-23 |
| IGF-1 | rs41316003 | 9 | 5126343 | G | A | 0.006 | -0.096 | 0.016 | 2.17E-09 |
| IGF-1 | rs11557154 | 9 | 34107505 | C | T | 0.127 | 0.022 | 0.004 | 4.21E-10 |
| IGF-1 | rs41277821 | 9 | 109689972 | C | T | 0.018 | 0.062 | 0.009 | 8.74E-12 |
| IGF-1 | rs3842752 | 11 | 2181073 | G | A | 0.228 | 0.060 | 0.003 | 3.30E-98 |
| IGF-1 | rs34312154 | 11 | 47470345 | G | A | 0.110 | 0.041 | 0.004 | 6.19E-26 |
| IGF-1 | rs11230983 | 11 | 55541284 | G | A | 0.130 | 0.033 | 0.004 | 1.28E-19 |
| IGF-1 | rs3759301 | 12 | 32135441 | T | C | 0.056 | 0.038 | 0.005 | 1.50E-13 |
| IGF-1 | rs78607331 | 12 | 57648644 | C | T | 0.045 | -0.036 | 0.006 | 5.85E-10 |
| IGF-1 | rs118092776 | 12 | 103306579 | C | T | 0.001 | 0.257 | 0.042 | 1.15E-09 |
| IGF-1 | rs1800574 | 12 | 121416864 | C | T | 0.029 | 0.145 | 0.007 | 5.13E-94 |
| IGF-1 | rs36215895 | 14 | 64676751 | C | T | 0.010 | -0.082 | 0.013 | 6.16E-11 |
| IGF-1 | rs28929474 | 14 | 94844947 | C | T | 0.020 | -0.061 | 0.009 | 1.29E-12 |
| IGF-1 | rs202077402 | 15 | 42982090 | A | G | 0.003 | -0.123 | 0.020 | 1.59E-09 |
| IGF-1 | rs55707100 | 15 | 43820717 | C | T | 0.025 | -0.149 | 0.008 | 5.17E-83 |
| IGF-1 | rs143076454 | 16 | 921179 | G | A | 0.019 | -0.071 | 0.009 | 1.50E-15 |
| IGF-1 | rs121917877 | 16 | 1129586 | C | T | 0.001 | 0.343 | 0.046 | 1.38E-13 |
| IGF-1 | rs61746451 | 16 | 1718110 | C | T | 0.009 | -0.073 | 0.012 | 3.44E-09 |
| IGF-1 | rs2294619 | 16 | 1814440 | A | G | 0.183 | -0.038 | 0.003 | 9.05E-36 |
| IGF-1 | rs35816944 | 16 | 1828030 | G | A | 0.007 | -0.104 | 0.015 | 2.61E-12 |
| IGF-1 | rs34680334 | 16 | 1842454 | G | A | 0.001 | -0.148 | 0.020 | 4.42E-14 |
| IGF-1 | rs79075024 | 16 | 1997385 | C | G | 0.026 | 0.060 | 0.008 | 8.41E-16 |
| IGF-1 | rs3803800 | 17 | 7462969 | A | G | 0.211 | 0.018 | 0.003 | 9.96E-10 |
| IGF-1 | rs5388 | 17 | 61995170 | C | T | 0.010 | 0.277 | 0.012 | 1.51E-114 |
| IGF-1 | rs1801689 | 17 | 64210580 | A | C | 0.030 | 0.086 | 0.007 | 1.66E-34 |
| IGF-1 | rs17265513 | 20 | 39832628 | T | C | 0.200 | -0.021 | 0.003 | 3.44E-12 |
| IGF-1 | rs2738787 | 20 | 62328375 | A | G | 0.082 | -0.035 | 0.004 | 1.94E-15 |

Abbreviations: IGF-1, insulin-like growth factor-1; SNP, single nucleotide polymorphism; Chr, chromosome; REF, reference allele; ALT, alter allele; MAF, minor allele frequency; SE, standard error; SHBG, sex hormones-binding globulin.

| **Phenotype** | **SNP** | **Chr** | **Position** | **gene** | **ALT** | **REF** | **MAF** | **Male** | | | **Female** | | |
| --- | --- | --- | --- | --- | --- | --- | --- | --- | --- | --- | --- | --- | --- |
|  |  |  |  |  |  |  |  | **Beta** | **SE** | ***p*-value** | **Beta** | **SE** | ***p*-value** |
| Estradiol | rs112881196 | 2 | 31982811 | SRD5A2 | G | C | 0.039 | 0.30 | 0.03 | 8.52E-24 | 0.10 | 0.03 | 1.13E-03 |
| Estradiol | rs62142080 | 2 | 32182528 | *MEMO1/ SRD5A2* | C | T | 0.396 | -0.08 | 0.01 | 1.09E-12 | -0.01 | 0.01 | 1.63E-01 |
| Estradiol | rs7662029 | 4 | 69961912 | *UGT2B7* | G | A | 0.454 | -0.11 | 0.01 | 3.61E-18 | -0.03 | 0.01 | 9.45E-03 |
| Estradiol | rs1073548 | 5 | 36012617 | *UGT3A1* | G | T | 0.126 | 0.11 | 0.02 | 1.75E-08 | 0.05 | 0.02 | 5.28E-03 |
| Estradiol | rs45446698 | 7 | 99332948 | *CYP3A7* | G | A | 0.043 | -0.21 | 0.03 | 1.38E-09 | -0.21 | 0.03 | 7.62E-12 |
| Estradiol | rs657152 | 9 | 136139265 | *ABO* | A | G | 0.34 | -0.12 | 0.01 | 4.14E-18 | -0.05 | 0.01 | 3.32E-04 |
| Estradiol | rs56196860 | 12 | 2908330 | *FKBP4* | A | C | 0.031 | 0.23 | 0.03 | 4.11E-12 | -0.06 | 0.03 | 6.56E-02 |
| Estradiol | rs11160915 | 14 | 106514653 | *IGHV3-7* | A | G | 0.444 | -0.16 | 0.01 | 3.08E-35 | -0.05 | 0.01 | 1.06E-04 |
| Estradiol | rs28892005 | 15 | 51519945 | *CYP19A1* | A | G | 0.35 | -0.23 | 0.01 | 1.01E-60 | -0.04 | 0.01 | 2.27E-03 |
| Estradiol | rs3751591 | 15 | 51606710 | *CYP19A1* | G | A | 0.167 | 0.12 | 0.02 | 1.73E-10 | 0.00 | 0.02 | 8.96E-01 |
| Estradiol | rs62059839 | 17 | 7533015 | *SHBG* | T | C | 0.262 | 0.09 | 0.01 | 8.53E-11 | 0.04 | 0.01 | 1.84E-03 |
| Estradiol | rs113047993 | 18 | 20585399 | *RBBP8* | T | C | 0.068 | -0.15 | 0.03 | 1.47E-08 | -0.02 | 0.02 | 3.37E-01 |
| Estradiol | rs62129966 | 19 | 48374950 | *SULT2A1* | A | G | 0.164 | 0.11 | 0.02 | 2.18E-10 | 0.01 | 0.02 | 4.56E-01 |
| Estradiol | rs16991615 | 20 | 5948227 | *MCM8* | A | G | 0.065 | 0.04 | 0.03 | 1.01E-01 | 0.13 | 0.02 | 4.67E-08 |
| Estradiol | rs5933688 | X | 8880680 | *FAM9A* | G | A | 0.275 | 0.11 | 0.01 | 7.49E-30 | 0.01 | 0.01 | 3.48E-01 |
| Estradiol | rs114255570 | X | 67005508 | *AR* | A | G | 0.077 | -0.11 | 0.02 | 5.60E-09 | 0.00 | 0.02 | 9.19E-01 |

**supplementary file 1b. Genetic instruments for estradiol in male and female**

Abbreviations: SNP, single nucleotide polymorphism; Chr, chromosome; ALT, alter allele; REF, reference allele; MAF, minor allele frequency; SE, standard error.

**supplementary file 1c. Genetic instruments for testosterone in male**

| **Phenotype** | **SNP** | **Chr** | **Position** | **ALT** | **REF** | **EAF** | **Beta** | **SE** | ***p*-value** |
| --- | --- | --- | --- | --- | --- | --- | --- | --- | --- |
| Testosterone | rs36086195 | 1 | 16510894 | T | C | 0.579 | 0.019 | 0.003 | 1.70E-09 |
| Testosterone | rs66844552 | 1 | 25802056 | A | G | 0.598 | 0.017 | 0.003 | 3.30E-08 |
| Testosterone | rs114165349 | 1 | 27021913 | G | C | 0.977 | 0.148 | 0.01 | 6.70E-51 |
| Testosterone | rs3768321 | 1 | 40035928 | G | T | 0.803 | 0.031 | 0.004 | 5.40E-15 |
| Testosterone | rs9970140 | 1 | 61684288 | G | A | 0.076 | 0.04 | 0.006 | 6.80E-12 |
| Testosterone | rs6676846 | 1 | 92942352 | A | G | 0.794 | 0.03 | 0.004 | 1.20E-15 |
| Testosterone | rs12406721 | 1 | 107563243 | T | G | 0.343 | 0.033 | 0.003 | 2.30E-28 |
| Testosterone | rs267733 | 1 | 150958836 | A | G | 0.839 | 0.023 | 0.004 | 1.80E-08 |
| Testosterone | rs34702488 | 1 | 163256609 | A | T | 0.176 | 0.026 | 0.004 | 1.70E-11 |
| Testosterone | rs12125882 | 1 | 172141403 | A | T | 0.579 | 0.017 | 0.003 | 3.70E-08 |
| Testosterone | rs35737316 | 1 | 204161534 | T | C | 0.246 | 0.036 | 0.004 | 3.50E-27 |
| Testosterone | rs10864086 | 1 | 214318748 | C | A | 0.256 | 0.022 | 0.003 | 5.30E-11 |
| Testosterone | rs12470971 | 2 | 11725241 | A | G | 0.338 | 0.017 | 0.003 | 2.60E-08 |
| Testosterone | rs11096640 | 2 | 20354039 | C | A | 0.515 | 0.017 | 0.003 | 6.40E-10 |
| Testosterone | rs1260326 | 2 | 27730940 | C | T | 0.605 | 0.062 | 0.003 | 2.70E-91 |
| Testosterone | rs113017476 | 2 | 31989359 | A | G | 0.039 | 0.19 | 0.008 | 7.00E-134 |
| Testosterone | rs6736913 | 2 | 42510018 | A | G | 0.021 | 0.062 | 0.01 | 1.20E-08 |
| Testosterone | rs12614829 | 2 | 64893183 | T | C | 0.207 | 0.03 | 0.004 | 6.10E-17 |
| Testosterone | rs2540945 | 2 | 65289825 | G | A | 0.363 | 0.018 | 0.003 | 8.00E-09 |
| Testosterone | rs6750410 | 2 | 70417730 | A | G | 0.935 | 0.042 | 0.006 | 5.20E-12 |
| Testosterone | rs10192634 | 2 | 180500950 | T | C | 0.269 | 0.023 | 0.003 | 2.00E-11 |
| Testosterone | rs2551641 | 2 | 208410267 | C | T | 0.189 | 0.022 | 0.004 | 7.20E-09 |
| Testosterone | rs2012736 | 2 | 234622379 | C | A | 0.918 | 0.048 | 0.006 | 5.10E-19 |
| Testosterone | rs6792725 | 3 | 24520283 | G | A | 0.694 | 0.019 | 0.003 | 3.20E-09 |
| Testosterone | rs7610366 | 3 | 28810588 | T | C | 0.726 | 0.021 | 0.003 | 2.20E-11 |
| Testosterone | rs112765699 | 3 | 61130525 | A | G | 0.015 | 0.075 | 0.013 | 2.20E-08 |
| Testosterone | rs66956368 | 3 | 61279726 | T | A | 0.703 | 0.03 | 0.004 | 2.10E-19 |
| Testosterone | rs55869022 | 3 | 61656512 | C | G | 0.868 | 0.027 | 0.005 | 2.00E-09 |
| Testosterone | rs34040779 | 3 | 107235109 | T | C | 0.924 | 0.034 | 0.006 | 5.30E-09 |
| Testosterone | rs645040 | 3 | 135926622 | G | T | 0.227 | 0.043 | 0.004 | 1.50E-35 |
| Testosterone | rs6766859 | 3 | 138055136 | T | C | 0.628 | 0.031 | 0.003 | 1.00E-21 |
| Testosterone | rs61762319 | 3 | 154801978 | G | A | 0.029 | 0.061 | 0.009 | 1.40E-11 |
| Testosterone | rs59194935 | 3 | 172147239 | A | G | 0.432 | 0.02 | 0.003 | 3.70E-11 |
| Testosterone | rs13074711 | 3 | 172267803 | T | C | 0.884 | 0.028 | 0.005 | 3.00E-09 |
| Testosterone | rs13108218 | 4 | 3443931 | A | G | 0.383 | 0.035 | 0.003 | 1.90E-29 |
| Testosterone | rs1203109 | 4 | 3455362 | C | T | 0.227 | 0.024 | 0.004 | 2.00E-11 |
| Testosterone | rs7679843 | 4 | 22028079 | G | C | 0.095 | 0.043 | 0.005 | 1.20E-17 |
| Testosterone | rs7696472 | 4 | 69538180 | A | G | 0.476 | 0.038 | 0.003 | 1.50E-39 |
| Testosterone | rs10028954 | 4 | 70466280 | C | T | 0.585 | 0.019 | 0.003 | 2.60E-09 |
| Testosterone | rs1441911 | 4 | 77193545 | G | T | 0.783 | 0.022 | 0.004 | 5.90E-09 |
| Testosterone | rs17408832 | 4 | 87222696 | C | G | 0.622 | 0.019 | 0.003 | 7.90E-10 |
| Testosterone | rs11735092 | 4 | 88226231 | T | C | 0.562 | 0.037 | 0.003 | 9.30E-35 |
| Testosterone | rs1154401 | 4 | 100009738 | G | C | 0.338 | 0.028 | 0.003 | 2.90E-18 |
| Testosterone | rs201814405 | 4 | 104174307 | A | T | 0.152 | 0.034 | 0.005 | 3.50E-13 |
| Testosterone | rs114816312 | 4 | 110638824 | T | C | 0.007 | 0.119 | 0.018 | 2.00E-11 |
| Testosterone | rs11099675 | 4 | 148985104 | T | C | 0.248 | 0.021 | 0.004 | 8.80E-10 |
| Testosterone | rs60701 | 5 | 10733776 | C | T | 0.257 | 0.019 | 0.003 | 4.30E-08 |
| Testosterone | rs7735249 | 5 | 53310139 | C | G | 0.887 | 0.028 | 0.005 | 1.80E-09 |
| Testosterone | rs40270 | 5 | 55804552 | A | C | 0.228 | 0.022 | 0.004 | 2.40E-09 |
| Testosterone | rs112530420 | 5 | 95871370 | C | T | 0.166 | 0.026 | 0.004 | 5.30E-10 |
| Testosterone | rs329122 | 5 | 133864599 | G | A | 0.581 | 0.018 | 0.003 | 2.90E-09 |
| Testosterone | rs6870458 | 5 | 137818916 | T | G | 0.549 | 0.023 | 0.003 | 4.10E-13 |
| Testosterone | rs1349359 | 5 | 165901446 | A | G | 0.438 | 0.017 | 0.003 | 2.10E-08 |
| Testosterone | rs9461224 | 6 | 25936402 | T | G | 0.397 | 0.018 | 0.003 | 4.00E-10 |
| Testosterone | rs2016572 | 6 | 31458983 | G | A | 0.332 | 0.026 | 0.003 | 5.80E-12 |
| Testosterone | rs543504257 | 6 | 32571403 | A | C | 0.506 | 0.024 | 0.003 | 1.30E-11 |
| Testosterone | rs6939861 | 6 | 41703041 | G | A | 0.738 | 0.025 | 0.003 | 1.70E-12 |
| Testosterone | rs62415384 | 6 | 43261767 | G | C | 0.548 | 0.016 | 0.003 | 2.60E-08 |
| Testosterone | rs1933801 | 6 | 105365725 | T | C | 0.678 | 0.038 | 0.003 | 7.80E-33 |
| Testosterone | rs9383605 | 6 | 152354561 | A | T | 0.705 | 0.019 | 0.003 | 2.40E-09 |
| Testosterone | rs7773995 | 6 | 154382367 | C | T | 0.831 | 0.024 | 0.004 | 1.20E-09 |
| Testosterone | rs9986829 | 7 | 15019259 | A | G | 0.507 | 0.041 | 0.003 | 5.20E-45 |
| Testosterone | rs1708302 | 7 | 28198677 | T | C | 0.502 | 0.018 | 0.003 | 2.60E-09 |
| Testosterone | rs10279715 | 7 | 40870935 | A | G | 0.538 | 0.018 | 0.003 | 9.90E-10 |
| Testosterone | rs1229498 | 7 | 81568750 | T | G | 0.278 | 0.019 | 0.003 | 6.50E-09 |
| Testosterone | rs7015 | 7 | 97920623 | G | A | 0.815 | 0.056 | 0.004 | 7.60E-49 |
| Testosterone | rs11772470 | 7 | 99181096 | A | G | 0.148 | 0.03 | 0.004 | 7.70E-14 |
| Testosterone | rs4841133 | 8 | 9183664 | G | A | 0.909 | 0.031 | 0.005 | 8.90E-10 |
| Testosterone | rs7835492 | 8 | 21089517 | C | T | 0.147 | 0.025 | 0.004 | 1.10E-09 |
| Testosterone | rs4871844 | 8 | 22879734 | C | T | 0.344 | 0.017 | 0.003 | 3.10E-08 |
| Testosterone | rs10958704 | 8 | 38328302 | A | G | 0.598 | 0.02 | 0.003 | 2.70E-11 |
| Testosterone | rs12543287 | 8 | 42334511 | C | G | 0.371 | 0.02 | 0.003 | 9.80E-11 |
| Testosterone | rs7844586 | 8 | 61782304 | C | T | 0.751 | 0.02 | 0.004 | 1.10E-08 |
| Testosterone | rs55867305 | 8 | 77884459 | G | A | 0.749 | 0.033 | 0.003 | 1.80E-21 |
| Testosterone | rs7824394 | 8 | 81292599 | C | A | 0.641 | 0.024 | 0.003 | 1.20E-13 |
| Testosterone | rs76767219 | 8 | 81426196 | A | C | 0.035 | 0.055 | 0.008 | 2.10E-12 |
| Testosterone | rs34955534 | 8 | 81710349 | G | A | 0.9 | 0.042 | 0.005 | 4.10E-16 |
| Testosterone | rs7828742 | 8 | 116960729 | A | G | 0.4 | 0.017 | 0.003 | 2.50E-08 |
| Testosterone | rs2721195 | 8 | 145677011 | T | C | 0.475 | 0.02 | 0.003 | 7.50E-11 |
| Testosterone | rs12336359 | 9 | 4129657 | C | G | 0.406 | 0.019 | 0.003 | 1.70E-09 |
| Testosterone | rs112107457 | 9 | 19103774 | T | C | 0.132 | 0.029 | 0.005 | 1.00E-10 |
| Testosterone | rs3808869 | 9 | 34622389 | C | A | 0.521 | 0.017 | 0.003 | 5.40E-09 |
| Testosterone | rs199950405 | 9 | 83271419 | C | G | 0.259 | 0.019 | 0.004 | 4.20E-08 |
| Testosterone | rs10868080 | 9 | 86626769 | T | A | 0.255 | 0.036 | 0.003 | 1.20E-27 |
| Testosterone | rs41310053 | 9 | 88940359 | T | C | 0.006 | 0.112 | 0.02 | 3.90E-08 |
| Testosterone | rs2090409 | 9 | 108967088 | C | A | 0.684 | 0.026 | 0.003 | 2.20E-16 |
| Testosterone | rs10982156 | 9 | 117088064 | A | T | 0.07 | 0.037 | 0.006 | 1.40E-09 |
| Testosterone | rs10982192 | 9 | 117149417 | T | C | 0.222 | 0.024 | 0.004 | 6.30E-11 |
| Testosterone | rs13289095 | 9 | 131466489 | G | T | 0.855 | 0.021 | 0.004 | 4.00E-08 |
| Testosterone | rs35182096 | 9 | 137268682 | C | T | 0.256 | 0.023 | 0.004 | 1.30E-10 |
| Testosterone | rs79717793 | 10 | 5262267 | G | A | 0.845 | 0.049 | 0.004 | 9.10E-36 |
| Testosterone | rs77044968 | 10 | 63657706 | C | T | 0.061 | 0.037 | 0.006 | 2.30E-09 |
| Testosterone | rs34131245 | 10 | 66174554 | C | A | 0.906 | 0.03 | 0.005 | 3.30E-08 |
| Testosterone | rs7912521 | 10 | 67262089 | C | T | 0.415 | 0.049 | 0.003 | 2.60E-61 |
| Testosterone | rs2862954 | 10 | 101912064 | C | T | 0.5 | 0.018 | 0.003 | 5.80E-09 |
| Testosterone | rs7915430 | 10 | 121660465 | T | G | 0.798 | 0.025 | 0.004 | 2.30E-12 |
| Testosterone | rs2957683 | 11 | 10352175 | C | G | 0.528 | 0.016 | 0.003 | 5.90E-09 |
| Testosterone | rs4757142 | 11 | 13325695 | G | A | 0.391 | 0.018 | 0.003 | 6.40E-09 |
| Testosterone | rs10832570 | 11 | 16249510 | A | G | 0.612 | 0.025 | 0.003 | 1.80E-16 |
| Testosterone | rs1994721 | 11 | 29204531 | G | A | 0.852 | 0.049 | 0.004 | 1.90E-32 |
| Testosterone | rs145843487 | 11 | 29323066 | G | C | 0.981 | 0.076 | 0.011 | 3.90E-12 |
| Testosterone | rs11607114 | 11 | 48151287 | C | G | 0.844 | 0.025 | 0.004 | 4.20E-10 |
| Testosterone | rs10750766 | 11 | 65473798 | C | A | 0.289 | 0.022 | 0.003 | 1.60E-11 |
| Testosterone | rs631695 | 11 | 69283303 | T | G | 0.417 | 0.024 | 0.003 | 7.20E-17 |
| Testosterone | rs12787293 | 11 | 72394147 | G | A | 0.458 | 0.02 | 0.003 | 8.00E-11 |
| Testosterone | rs12796488 | 11 | 94131557 | C | A | 0.823 | 0.041 | 0.004 | 2.00E-26 |
| Testosterone | rs4754839 | 11 | 102157900 | A | G | 0.562 | 0.018 | 0.003 | 1.10E-08 |
| Testosterone | rs10892924 | 11 | 122773715 | T | A | 0.568 | 0.032 | 0.003 | 7.60E-26 |
| Testosterone | rs618888 | 11 | 125081521 | T | G | 0.283 | 0.023 | 0.003 | 1.30E-12 |
| Testosterone | rs56196860 | 12 | 2908330 | A | C | 0.032 | 0.301 | 0.009 | 5.10E-276 |
| Testosterone | rs61922185 | 12 | 21039679 | T | G | 0.321 | 0.021 | 0.003 | 1.80E-11 |
| Testosterone | rs73079476 | 12 | 21343833 | A | C | 0.849 | 0.054 | 0.004 | 4.20E-40 |
| Testosterone | rs12320328 | 12 | 25408464 | A | G | 0.915 | 0.043 | 0.005 | 2.30E-16 |
| Testosterone | rs540730 | 12 | 57807114 | T | C | 0.245 | 0.03 | 0.004 | 3.70E-19 |
| Testosterone | rs2583948 | 12 | 66194613 | A | G | 0.903 | 0.035 | 0.005 | 3.00E-11 |
| Testosterone | rs191591035 | 12 | 99868285 | G | C | 0.997 | 0.222 | 0.035 | 2.50E-10 |
| Testosterone | rs61755050 | 12 | 100926308 | T | C | 0.994 | 0.211 | 0.02 | 1.30E-30 |
| Testosterone | rs7314285 | 12 | 111522026 | G | T | 0.068 | 0.039 | 0.006 | 8.80E-12 |
| Testosterone | rs3809272 | 12 | 111800258 | G | A | 0.697 | 0.02 | 0.003 | 3.90E-10 |
| Testosterone | rs12810788 | 12 | 116196322 | G | A | 0.201 | 0.025 | 0.004 | 1.50E-10 |
| Testosterone | rs2393775 | 12 | 121424574 | A | G | 0.623 | 0.026 | 0.003 | 2.70E-17 |
| Testosterone | rs6486542 | 12 | 130952209 | C | T | 0.57 | 0.021 | 0.003 | 1.30E-11 |
| Testosterone | rs7997628 | 13 | 95217852 | A | T | 0.35 | 0.017 | 0.003 | 2.80E-08 |
| Testosterone | rs2038695 | 13 | 100559123 | C | A | 0.449 | 0.021 | 0.003 | 1.10E-12 |
| Testosterone | rs3742223 | 13 | 112725196 | T | C | 0.904 | 0.03 | 0.005 | 2.80E-10 |
| Testosterone | rs139713000 | 14 | 23731855 | CT | C | 0.196 | 0.028 | 0.004 | 1.30E-15 |
| Testosterone | rs11621792 | 14 | 24871926 | C | T | 0.546 | 0.018 | 0.003 | 6.70E-09 |
| Testosterone | rs72681869 | 14 | 50655357 | C | G | 0.011 | 0.103 | 0.015 | 3.70E-14 |
| Testosterone | rs2239222 | 14 | 73011885 | G | A | 0.349 | 0.021 | 0.003 | 1.80E-10 |
| Testosterone | rs72721770 | 14 | 74204686 | G | C | 0.652 | 0.019 | 0.003 | 1.00E-09 |
| Testosterone | rs1812755 | 14 | 90007637 | T | C | 0.8 | 0.026 | 0.004 | 1.50E-11 |
| Testosterone | rs145602600 | 14 | 94838202 | T | C | 0.005 | 0.133 | 0.021 | 8.20E-11 |
| Testosterone | rs28929474 | 14 | 94844947 | T | C | 0.02 | 0.222 | 0.011 | 1.20E-95 |
| Testosterone | rs17580 | 14 | 94847262 | A | T | 0.048 | 0.049 | 0.007 | 1.60E-12 |
| Testosterone | rs7143218 | 14 | 100797255 | C | A | 0.269 | 0.025 | 0.003 | 4.90E-14 |
| Testosterone | rs45490496 | 14 | 105272678 | A | T | 0.387 | 0.021 | 0.003 | 2.60E-11 |
| Testosterone | rs55707100 | 15 | 43820717 | C | T | 0.975 | 0.111 | 0.01 | 1.80E-33 |
| Testosterone | rs140357247 | 15 | 52734389 | C | G | 0.998 | 0.241 | 0.038 | 7.20E-10 |
| Testosterone | rs79391862 | 15 | 53739426 | A | C | 0.986 | 0.157 | 0.013 | 5.60E-38 |
| Testosterone | rs12910403 | 15 | 57073464 | G | C | 0.634 | 0.018 | 0.003 | 2.30E-08 |
| Testosterone | rs7182912 | 15 | 89038812 | G | T | 0.809 | 0.021 | 0.004 | 3.90E-09 |
| Testosterone | rs7166920 | 15 | 96219503 | A | G | 0.501 | 0.02 | 0.003 | 1.10E-10 |
| Testosterone | rs56332871 | 15 | 96714816 | A | C | 0.272 | 0.047 | 0.003 | 1.70E-47 |
| Testosterone | rs841194 | 16 | 4667690 | G | A | 0.822 | 0.027 | 0.004 | 6.70E-11 |
| Testosterone | rs12445820 | 16 | 11866703 | G | C | 0.148 | 0.025 | 0.004 | 8.00E-09 |
| Testosterone | rs2764772 | 16 | 20060653 | A | T | 0.334 | 0.032 | 0.003 | 2.50E-24 |
| Testosterone | rs1421085 | 16 | 53800954 | T | C | 0.595 | 0.022 | 0.003 | 1.20E-11 |
| Testosterone | rs42945 | 16 | 58545426 | A | G | 0.488 | 0.016 | 0.003 | 4.30E-08 |
| Testosterone | rs79488654 | 16 | 82131688 | G | T | 0.999 | 0.235 | 0.041 | 7.90E-09 |
| Testosterone | rs4782568 | 16 | 83980529 | G | C | 0.452 | 0.025 | 0.003 | 7.90E-17 |
| Testosterone | rs12926107 | 16 | 88004092 | A | G | 0.542 | 0.024 | 0.003 | 7.10E-16 |
| Testosterone | rs550628400 | 17 | 1639795 | G | A | 0.006 | 0.134 | 0.02 | 8.60E-11 |
| Testosterone | rs4525526 | 17 | 1650125 | C | T | 0.219 | 0.033 | 0.004 | 2.50E-20 |
| Testosterone | rs1799941 | 17 | 7533423 | A | G | 0.261 | 0.197 | 0.003 | 9.1E-742 |
| Testosterone | rs6258 | 17 | 7534678 | C | T | 0.993 | 0.718 | 0.018 | 6.4E-347 |
| Testosterone | rs56853305 | 17 | 27648542 | G | A | 0.859 | 0.027 | 0.004 | 4.40E-10 |
| Testosterone | rs2905801 | 17 | 29524974 | T | C | 0.706 | 0.029 | 0.003 | 3.60E-19 |
| Testosterone | rs650558 | 17 | 40721042 | C | T | 0.752 | 0.022 | 0.003 | 1.60E-10 |
| Testosterone | rs62062271 | 17 | 44091988 | C | T | 0.228 | 0.031 | 0.004 | 5.40E-18 |
| Testosterone | rs28394864 | 17 | 47450775 | G | A | 0.539 | 0.053 | 0.003 | 1.50E-72 |
| Testosterone | rs2306216 | 17 | 73240559 | A | G | 0.168 | 0.027 | 0.004 | 9.20E-12 |
| Testosterone | rs7216664 | 17 | 73825664 | G | A | 0.362 | 0.019 | 0.003 | 2.40E-10 |
| Testosterone | rs62076019 | 17 | 79481003 | G | T | 0.601 | 0.016 | 0.003 | 4.10E-08 |
| Testosterone | rs2668776 | 18 | 44750365 | C | T | 0.469 | 0.023 | 0.003 | 6.80E-14 |
| Testosterone | rs1624295 | 19 | 2792034 | A | G | 0.293 | 0.033 | 0.003 | 7.20E-24 |
| Testosterone | rs8107967 | 19 | 7972615 | G | A | 0.568 | 0.019 | 0.003 | 1.70E-10 |
| Testosterone | rs10421262 | 19 | 14172951 | T | G | 0.576 | 0.021 | 0.003 | 1.70E-12 |
| Testosterone | rs202200760 | 19 | 17346854 | C | G | 0.039 | 0.12 | 0.009 | 8.60E-48 |
| Testosterone | rs35824797 | 19 | 19456264 | C | T | 0.921 | 0.044 | 0.006 | 1.70E-15 |
| Testosterone | rs34851490 | 19 | 46384554 | G | A | 0.116 | 0.048 | 0.005 | 1.80E-23 |
| Testosterone | rs11671304 | 19 | 47564643 | C | T | 0.329 | 0.016 | 0.003 | 4.40E-08 |
| Testosterone | rs6073431 | 20 | 43040569 | T | C | 0.531 | 0.031 | 0.003 | 5.00E-24 |
| Testosterone | rs1058319 | 20 | 62374389 | T | C | 0.135 | 0.024 | 0.005 | 1.50E-08 |
| Testosterone | rs575146 | 22 | 24295074 | G | A | 0.398 | 0.018 | 0.003 | 1.10E-08 |
| Testosterone | rs1033667 | 22 | 29130300 | T | C | 0.3 | 0.023 | 0.003 | 9.30E-13 |
| Testosterone | rs738409 | 22 | 44324727 | G | C | 0.216 | 0.051 | 0.004 | 4.30E-43 |
| Testosterone | rs11703376 | 22 | 49678713 | T | C | 0.27 | 0.034 | 0.003 | 2.70E-23 |
| Testosterone | rs112265145 | 23 | 8906893 | C | A | 0.267 | 0.098 | 0.002 | 1.1E-372 |
| Testosterone | rs146447930 | 23 | 63167507 | C | T | 0.986 | 0.071 | 0.009 | 2.50E-16 |
| Testosterone | rs74805556 | 23 | 65779640 | T | C | 0.724 | 0.04 | 0.002 | 1.40E-60 |
| Testosterone | rs72630041 | 23 | 71399338 | G | A | 0.192 | 0.018 | 0.003 | 1.90E-11 |
| Testosterone | rs881090 | 23 | 109820220 | T | G | 0.391 | 0.063 | 0.002 | 5.90E-188 |
| Testosterone | rs7065171 | 23 | 133683590 | G | C | 0.654 | 0.018 | 0.002 | 1.70E-16 |

Abbreviations: SNP, single nucleotide polymorphism; Chr, chromosome; REF, reference allele; ALT, alter allele; EAF, effect allele frequency; SE, standard error.

**supplementary file 1d. Genetic instruments for testosterone in female**

| **Phenotype** | **SNP** | **Chr** | **Position** | **ALT** | **REF** | **EAF** | **Beta** | **SE** | ***p*-value** |
| --- | --- | --- | --- | --- | --- | --- | --- | --- | --- |
| Testosterone | rs571084788 | 1 | 7843711 | T | C | 0.807 | 0.024 | 0.004 | 1.40E-11 |
| Testosterone | rs10799713 | 1 | 22102328 | G | C | 0.212 | 0.027 | 0.003 | 9.20E-16 |
| Testosterone | rs7530117 | 1 | 31263360 | T | C | 0.369 | 0.018 | 0.003 | 1.30E-09 |
| Testosterone | rs4453027 | 1 | 41453453 | G | T | 0.575 | 0.023 | 0.003 | 1.40E-15 |
| Testosterone | rs1278526 | 1 | 50891213 | A | C | 0.524 | 0.019 | 0.003 | 2.10E-12 |
| Testosterone | rs7529520 | 1 | 57016477 | C | G | 0.486 | 0.018 | 0.003 | 6.20E-12 |
| Testosterone | rs505237 | 1 | 66738623 | G | A | 0.616 | 0.016 | 0.003 | 3.70E-08 |
| Testosterone | rs111882448 | 1 | 68846740 | CA | C | 0.351 | 0.017 | 0.003 | 3.80E-09 |
| Testosterone | rs4294422 | 1 | 93104655 | G | A | 0.359 | 0.019 | 0.003 | 6.80E-11 |
| Testosterone | rs6684361 | 1 | 101737743 | C | T | 0.307 | 0.076 | 0.003 | 7.40E-141 |
| Testosterone | rs3032555 | 1 | 107607035 | T | TCAG | 0.342 | 0.018 | 0.003 | 4.40E-09 |
| Testosterone | rs7519368 | 1 | 113150644 | T | A | 0.727 | 0.033 | 0.003 | 3.10E-25 |
| Testosterone | rs72693130 | 1 | 120013323 | A | G | 0.063 | 0.031 | 0.006 | 2.90E-08 |
| Testosterone | rs58175144 | 1 | 150839698 | CA | C | 0.303 | 0.021 | 0.003 | 3.50E-12 |
| Testosterone | rs1870940 | 1 | 154984363 | G | A | 0.727 | 0.025 | 0.003 | 4.90E-17 |
| Testosterone | rs76830943 | 1 | 156009127 | T | C | 0.833 | 0.024 | 0.004 | 3.00E-10 |
| Testosterone | rs12564492 | 1 | 168234645 | A | G | 0.703 | 0.02 | 0.003 | 3.80E-11 |
| Testosterone | rs2062479 | 1 | 197868281 | C | G | 0.524 | 0.016 | 0.003 | 1.80E-08 |
| Testosterone | rs17043570 | 1 | 216905301 | T | C | 0.159 | 0.025 | 0.004 | 4.10E-12 |
| Testosterone | rs12078363 | 1 | 218537310 | T | C | 0.681 | 0.028 | 0.003 | 1.00E-21 |
| Testosterone | rs10910476 | 1 | 234734956 | C | T | 0.444 | 0.017 | 0.003 | 1.10E-09 |
| Testosterone | rs34269793 | 2 | 12555164 | C | T | 0.053 | 0.064 | 0.006 | 2.30E-25 |
| Testosterone | rs3771243 | 2 | 20412227 | A | G | 0.39 | 0.029 | 0.003 | 1.40E-26 |
| Testosterone | rs11892043 | 2 | 24990082 | A | G | 0.728 | 0.022 | 0.003 | 2.40E-11 |
| Testosterone | rs1260326 | 2 | 27730940 | C | T | 0.607 | 0.037 | 0.003 | 4.70E-39 |
| Testosterone | rs2374456 | 2 | 43271621 | G | C | 0.584 | 0.024 | 0.003 | 9.30E-17 |
| Testosterone | rs7575635 | 2 | 43515427 | C | T | 0.806 | 0.038 | 0.004 | 1.40E-27 |
| Testosterone | rs3136354 | 2 | 48031372 | C | T | 0.501 | 0.024 | 0.003 | 3.50E-18 |
| Testosterone | rs11125180 | 2 | 48942282 | A | T | 0.909 | 0.033 | 0.005 | 5.40E-12 |
| Testosterone | rs7573187 | 2 | 62536671 | A | T | 0.535 | 0.019 | 0.003 | 1.30E-11 |
| Testosterone | rs1009360 | 2 | 65276049 | C | T | 0.416 | 0.018 | 0.003 | 1.50E-09 |
| Testosterone | rs10865479 | 2 | 86095432 | T | C | 0.715 | 0.022 | 0.003 | 2.40E-13 |
| Testosterone | rs113247979 | 2 | 103338672 | T | C | 0.993 | 0.14 | 0.017 | 6.60E-17 |
| Testosterone | rs590097 | 2 | 111934107 | G | T | 0.646 | 0.061 | 0.003 | 1.60E-100 |
| Testosterone | rs62162863 | 2 | 112071459 | G | T | 0.577 | 0.023 | 0.003 | 1.10E-13 |
| Testosterone | rs10168169 | 2 | 112375425 | T | C | 0.812 | 0.033 | 0.004 | 5.10E-20 |
| Testosterone | rs59741822 | 2 | 136970398 | G | A | 0.923 | 0.033 | 0.005 | 7.00E-12 |
| Testosterone | rs58723250 | 2 | 178153430 | T | C | 0.2 | 0.035 | 0.004 | 2.20E-23 |
| Testosterone | rs36088520 | 2 | 197779590 | T | C | 0.104 | 0.028 | 0.005 | 5.70E-09 |
| Testosterone | rs873779 | 2 | 208773860 | C | T | 0.372 | 0.016 | 0.003 | 1.80E-09 |
| Testosterone | rs2011425 | 2 | 234627608 | T | G | 0.921 | 0.033 | 0.005 | 6.60E-11 |
| Testosterone | rs7618363 | 3 | 10545125 | C | G | 0.841 | 0.037 | 0.004 | 1.20E-21 |
| Testosterone | rs62231822 | 3 | 14423060 | C | T | 0.902 | 0.049 | 0.005 | 5.90E-26 |
| Testosterone | rs17201704 | 3 | 41105589 | T | C | 0.859 | 0.043 | 0.004 | 2.90E-28 |
| Testosterone | rs4067 | 3 | 51738256 | G | A | 0.855 | 0.024 | 0.004 | 1.70E-10 |
| Testosterone | rs13094915 | 3 | 52507719 | G | C | 0.632 | 0.016 | 0.003 | 2.00E-09 |
| Testosterone | rs9832502 | 3 | 72398979 | A | G | 0.249 | 0.017 | 0.003 | 3.80E-09 |
| Testosterone | rs167096 | 3 | 73874699 | T | G | 0.26 | 0.017 | 0.003 | 4.30E-10 |
| Testosterone | rs7633673 | 3 | 152084243 | G | A | 0.594 | 0.026 | 0.003 | 7.50E-20 |
| Testosterone | rs9850919 | 3 | 169177924 | C | T | 0.405 | 0.02 | 0.003 | 2.80E-13 |
| Testosterone | rs77822621 | 4 | 1008212 | T | C | 0.042 | 0.048 | 0.007 | 9.30E-14 |
| Testosterone | rs3849653 | 4 | 53860084 | A | T | 0.541 | 0.019 | 0.003 | 6.60E-12 |
| Testosterone | rs4632729 | 4 | 69946004 | A | G | 0.545 | 0.028 | 0.003 | 2.40E-22 |
| Testosterone | rs1229984 | 4 | 100239319 | C | T | 0.976 | 0.049 | 0.009 | 1.20E-08 |
| Testosterone | rs4586943 | 4 | 102366529 | A | C | 0.282 | 0.018 | 0.003 | 6.90E-09 |
| Testosterone | rs28484580 | 4 | 103423549 | G | A | 0.224 | 0.021 | 0.004 | 1.70E-08 |
| Testosterone | rs2903385 | 4 | 106094427 | A | G | 0.486 | 0.025 | 0.003 | 1.60E-18 |
| Testosterone | rs4245930 | 4 | 109038654 | G | A | 0.368 | 0.025 | 0.003 | 1.10E-19 |
| Testosterone | rs371162363 | 4 | 177614117 | G | A | 0.904 | 0.039 | 0.005 | 5.60E-15 |
| Testosterone | rs112694713 | 5 | 35247932 | A | G | 0.986 | 0.107 | 0.012 | 2.40E-19 |
| Testosterone | rs9687846 | 5 | 55861894 | G | A | 0.8 | 0.026 | 0.004 | 3.60E-12 |
| Testosterone | rs4431325 | 5 | 76461706 | T | C | 0.059 | 0.05 | 0.006 | 8.50E-18 |
| Testosterone | rs1119208 | 5 | 76488613 | C | T | 0.648 | 0.03 | 0.003 | 9.80E-26 |
| Testosterone | rs784420 | 5 | 77987524 | G | A | 0.289 | 0.039 | 0.003 | 1.20E-39 |
| Testosterone | rs11948639 | 5 | 122741746 | T | C | 0.434 | 0.017 | 0.003 | 3.20E-09 |
| Testosterone | rs12658172 | 5 | 124205385 | G | C | 0.842 | 0.052 | 0.004 | 5.80E-41 |
| Testosterone | rs13184921 | 5 | 127870642 | T | C | 0.753 | 0.025 | 0.003 | 7.60E-16 |
| Testosterone | rs3776299 | 5 | 142507651 | G | A | 0.549 | 0.017 | 0.003 | 3.60E-10 |
| Testosterone | rs13153019 | 5 | 176782218 | C | T | 0.249 | 0.024 | 0.003 | 1.50E-14 |
| Testosterone | rs75217853 | 6 | 1377048 | A | G | 0.098 | 0.035 | 0.005 | 1.30E-14 |
| Testosterone | rs267190 | 6 | 7842121 | G | T | 0.578 | 0.015 | 0.003 | 3.70E-08 |
| Testosterone | rs6904345 | 6 | 24697424 | T | C | 0.634 | 0.017 | 0.003 | 1.90E-08 |
| Testosterone | rs487624 | 6 | 25879539 | C | A | 0.568 | 0.019 | 0.003 | 1.40E-12 |
| Testosterone | rs2517582 | 6 | 30808762 | T | C | 0.384 | 0.028 | 0.003 | 1.60E-18 |
| Testosterone | rs184265581 | 6 | 31900754 | C | G | 0.012 | 0.132 | 0.013 | 6.40E-27 |
| Testosterone | rs1214761 | 6 | 43354431 | G | A | 0.679 | 0.031 | 0.003 | 6.20E-26 |
| Testosterone | rs2608652 | 6 | 52642768 | T | C | 0.525 | 0.015 | 0.003 | 1.30E-08 |
| Testosterone | rs1032388 | 6 | 119171866 | C | T | 0.779 | 0.054 | 0.003 | 2.50E-61 |
| Testosterone | rs577721086 | 6 | 127440047 | T | C | 0.95 | 0.055 | 0.007 | 5.80E-17 |
| Testosterone | rs2473140 | 6 | 144620688 | C | T | 0.09 | 0.029 | 0.005 | 7.20E-10 |
| Testosterone | rs287884 | 6 | 157124080 | A | T | 0.444 | 0.019 | 0.003 | 2.10E-11 |
| Testosterone | rs9457466 | 6 | 159224561 | A | T | 0.363 | 0.017 | 0.003 | 1.50E-09 |
| Testosterone | rs2344744 | 6 | 170588559 | G | T | 0.415 | 0.018 | 0.003 | 1.30E-09 |
| Testosterone | rs28612846 | 7 | 25950880 | G | A | 0.743 | 0.016 | 0.003 | 4.70E-08 |
| Testosterone | rs6460528 | 7 | 69218337 | T | C | 0.454 | 0.015 | 0.003 | 4.30E-08 |
| Testosterone | rs13229619 | 7 | 73030175 | A | G | 0.129 | 0.054 | 0.004 | 8.30E-39 |
| Testosterone | rs17853284 | 7 | 75610876 | C | T | 0.995 | 0.304 | 0.021 | 5.30E-49 |
| Testosterone | rs45446698 | 7 | 99332948 | T | G | 0.958 | 0.371 | 0.007 | 1.5E-635 |
| Testosterone | rs62621812 | 7 | 127015083 | A | G | 0.02 | 0.053 | 0.01 | 1.30E-08 |
| Testosterone | rs1872930 | 7 | 137801444 | T | C | 0.793 | 0.051 | 0.003 | 1.70E-54 |
| Testosterone | rs9638084 | 7 | 156311745 | G | A | 0.604 | 0.021 | 0.003 | 1.30E-13 |
| Testosterone | rs17362923 | 8 | 5546824 | G | C | 0.189 | 0.023 | 0.004 | 3.00E-11 |
| Testosterone | rs6997799 | 8 | 10606421 | C | A | 0.234 | 0.024 | 0.003 | 5.80E-13 |
| Testosterone | rs56109436 | 8 | 11543663 | G | C | 0.099 | 0.026 | 0.005 | 6.00E-09 |
| Testosterone | rs17053931 | 8 | 25417528 | A | G | 0.199 | 0.021 | 0.004 | 4.50E-09 |
| Testosterone | rs11782259 | 8 | 37532984 | A | G | 0.911 | 0.031 | 0.005 | 1.10E-10 |
| Testosterone | rs11778724 | 8 | 49549726 | C | A | 0.013 | 0.07 | 0.013 | 1.50E-08 |
| Testosterone | rs10108398 | 8 | 59440824 | G | A | 0.279 | 0.018 | 0.003 | 1.10E-08 |
| Testosterone | rs13269725 | 8 | 72459889 | A | G | 0.921 | 0.03 | 0.005 | 1.80E-08 |
| Testosterone | rs1660322 | 8 | 101277640 | T | C | 0.687 | 0.023 | 0.003 | 4.50E-15 |
| Testosterone | rs11774829 | 8 | 105978368 | A | T | 0.101 | 0.056 | 0.005 | 5.80E-32 |
| Testosterone | rs4736359 | 8 | 143987427 | T | G | 0.443 | 0.037 | 0.003 | 6.20E-40 |
| Testosterone | rs12683780 | 9 | 16252807 | A | C | 0.668 | 0.031 | 0.003 | 7.50E-26 |
| Testosterone | rs4961485 | 9 | 16360889 | T | C | 0.934 | 0.039 | 0.006 | 8.90E-14 |
| Testosterone | rs61237993 | 9 | 34130435 | A | G | 0.128 | 0.04 | 0.004 | 6.90E-23 |
| Testosterone | rs10821415 | 9 | 97713459 | C | A | 0.579 | 0.015 | 0.003 | 1.60E-08 |
| Testosterone | rs1547308 | 9 | 114603240 | C | T | 0.8 | 0.032 | 0.004 | 9.20E-21 |
| Testosterone | rs10817260 | 9 | 114828332 | C | T | 0.81 | 0.043 | 0.004 | 9.30E-35 |
| Testosterone | rs494242 | 9 | 136145118 | C | T | 0.659 | 0.017 | 0.003 | 1.70E-08 |
| Testosterone | rs35249079 | 10 | 1037737 | T | G | 0.531 | 0.022 | 0.003 | 1.40E-15 |
| Testosterone | rs36032941 | 10 | 5062752 | C | A | 0.704 | 0.063 | 0.003 | 3.00E-94 |
| Testosterone | rs1171617 | 10 | 61467182 | T | G | 0.767 | 0.052 | 0.003 | 5.50E-57 |
| Testosterone | rs10740131 | 10 | 65271488 | T | A | 0.473 | 0.028 | 0.003 | 2.10E-25 |
| Testosterone | rs674486 | 10 | 69819917 | C | T | 0.35 | 0.018 | 0.003 | 1.10E-08 |
| Testosterone | rs35199395 | 10 | 70983936 | C | G | 0.694 | 0.017 | 0.003 | 7.40E-09 |
| Testosterone | rs2147419 | 10 | 90759916 | T | G | 0.718 | 0.018 | 0.003 | 1.60E-09 |
| Testosterone | rs111328885 | 10 | 96823812 | C | A | 0.878 | 0.033 | 0.004 | 1.50E-14 |
| Testosterone | rs11191421 | 10 | 104623053 | C | G | 0.758 | 0.037 | 0.003 | 4.20E-31 |
| Testosterone | rs11191801 | 10 | 105532165 | A | C | 0.708 | 0.023 | 0.003 | 8.70E-16 |
| Testosterone | rs440150 | 11 | 2936657 | G | A | 0.093 | 0.026 | 0.005 | 2.20E-08 |
| Testosterone | rs11024458 | 11 | 18082483 | A | G | 0.728 | 0.019 | 0.003 | 2.30E-10 |
| Testosterone | rs10501081 | 11 | 27236061 | G | C | 0.138 | 0.022 | 0.004 | 1.30E-08 |
| Testosterone | rs11031005 | 11 | 30226356 | C | T | 0.143 | 0.033 | 0.004 | 7.20E-17 |
| Testosterone | rs1939769 | 11 | 62914202 | A | G | 0.066 | 0.083 | 0.006 | 1.30E-51 |
| Testosterone | rs35008345 | 11 | 64323613 | C | T | 0.995 | 0.183 | 0.019 | 1.20E-20 |
| Testosterone | rs312023 | 11 | 68097049 | G | A | 0.472 | 0.028 | 0.003 | 3.00E-24 |
| Testosterone | rs171021 | 11 | 72317557 | C | T | 0.703 | 0.038 | 0.003 | 4.20E-35 |
| Testosterone | rs11235688 | 11 | 72947934 | G | A | 0.583 | 0.022 | 0.003 | 3.70E-16 |
| Testosterone | rs75848431 | 11 | 123325111 | T | C | 0.152 | 0.032 | 0.004 | 4.20E-15 |
| Testosterone | rs76299412 | 11 | 128269098 | A | G | 0.153 | 0.035 | 0.004 | 2.00E-20 |
| Testosterone | rs56196860 | 12 | 2908330 | C | A | 0.969 | 0.058 | 0.008 | 3.70E-12 |
| Testosterone | rs881613 | 12 | 12738486 | A | G | 0.301 | 0.017 | 0.003 | 3.10E-08 |
| Testosterone | rs4149056 | 12 | 21331549 | C | T | 0.151 | 0.029 | 0.004 | 1.10E-14 |
| Testosterone | rs117913411 | 12 | 48254353 | T | A | 0.966 | 0.048 | 0.008 | 3.30E-10 |
| Testosterone | rs35801460 | 12 | 49180828 | G | A | 0.797 | 0.02 | 0.003 | 6.40E-09 |
| Testosterone | rs7977247 | 12 | 107259470 | T | C | 0.578 | 0.018 | 0.003 | 3.90E-11 |
| Testosterone | rs35427 | 12 | 115556307 | T | G | 0.617 | 0.017 | 0.003 | 3.40E-10 |
| Testosterone | rs1169289 | 12 | 121416622 | G | C | 0.44 | 0.019 | 0.003 | 2.50E-11 |
| Testosterone | rs837493 | 12 | 125075660 | G | A | 0.452 | 0.016 | 0.003 | 1.10E-08 |
| Testosterone | rs9506725 | 13 | 22314146 | T | C | 0.629 | 0.052 | 0.003 | 4.80E-73 |
| Testosterone | rs9552597 | 13 | 22690127 | A | G | 0.191 | 0.035 | 0.004 | 5.20E-24 |
| Testosterone | rs4943729 | 13 | 32353100 | A | C | 0.518 | 0.015 | 0.003 | 1.10E-08 |
| Testosterone | rs17764067 | 13 | 33728353 | G | A | 0.78 | 0.021 | 0.003 | 5.50E-10 |
| Testosterone | rs17245822 | 13 | 73131694 | C | A | 0.373 | 0.028 | 0.003 | 2.30E-23 |
| Testosterone | rs9599996 | 13 | 73203811 | T | G | 0.599 | 0.017 | 0.003 | 2.10E-10 |
| Testosterone | rs72660136 | 13 | 109960307 | T | C | 0.966 | 0.06 | 0.008 | 4.90E-16 |
| Testosterone | rs7342537 | 14 | 21555063 | G | A | 0.018 | 0.107 | 0.011 | 2.80E-24 |
| Testosterone | rs59397130 | 14 | 64898076 | G | A | 0.026 | 0.056 | 0.009 | 2.30E-10 |
| Testosterone | rs61987429 | 14 | 65606248 | C | T | 0.649 | 0.017 | 0.003 | 9.20E-10 |
| Testosterone | rs1314911 | 14 | 68698230 | A | G | 0.859 | 0.026 | 0.004 | 7.20E-10 |
| Testosterone | rs72731535 | 14 | 69237902 | G | A | 0.822 | 0.025 | 0.004 | 9.10E-12 |
| Testosterone | rs112635299 | 14 | 94838142 | G | T | 0.979 | 0.062 | 0.01 | 2.90E-11 |
| Testosterone | rs12436785 | 14 | 98550490 | C | T | 0.417 | 0.03 | 0.003 | 2.80E-26 |
| Testosterone | rs10147094 | 14 | 99727591 | A | G | 0.614 | 0.022 | 0.003 | 6.90E-16 |
| Testosterone | rs12893790 | 14 | 101178804 | G | C | 0.816 | 0.021 | 0.004 | 1.00E-09 |
| Testosterone | rs77432559 | 14 | 106875550 | G | C | 0.079 | 0.037 | 0.006 | 4.20E-11 |
| Testosterone | rs11638521 | 15 | 40360314 | T | C | 0.347 | 0.064 | 0.003 | 2.60E-110 |
| Testosterone | rs61661087 | 15 | 40720543 | C | T | 0.524 | 0.028 | 0.003 | 1.60E-24 |
| Testosterone | rs72738949 | 15 | 50668999 | T | A | 0.42 | 0.019 | 0.003 | 3.20E-12 |
| Testosterone | rs2113944 | 15 | 60977882 | T | C | 0.223 | 0.027 | 0.003 | 2.70E-15 |
| Testosterone | rs12708515 | 15 | 75454879 | G | C | 0.364 | 0.021 | 0.003 | 2.10E-14 |
| Testosterone | rs4464040 | 15 | 79840557 | C | T | 0.85 | 0.045 | 0.004 | 4.40E-32 |
| Testosterone | rs12900736 | 15 | 85558140 | C | T | 0.83 | 0.04 | 0.004 | 1.90E-27 |
| Testosterone | rs2074585 | 15 | 91009484 | A | G | 0.514 | 0.016 | 0.003 | 8.80E-09 |
| Testosterone | rs437115 | 16 | 4156423 | T | C | 0.565 | 0.028 | 0.003 | 4.20E-24 |
| Testosterone | rs8045779 | 16 | 12907061 | T | C | 0.836 | 0.026 | 0.004 | 1.40E-11 |
| Testosterone | rs8044588 | 16 | 67420328 | C | G | 0.089 | 0.028 | 0.005 | 1.50E-09 |
| Testosterone | rs58072681 | 16 | 81590541 | C | T | 0.07 | 0.108 | 0.006 | 7.90E-86 |
| Testosterone | rs187370584 | 17 | 2309972 | A | G | 0.987 | 0.066 | 0.012 | 1.50E-08 |
| Testosterone | rs9898480 | 17 | 6525861 | T | C | 0.569 | 0.018 | 0.003 | 9.80E-12 |
| Testosterone | rs62059839 | 17 | 7533015 | T | C | 0.261 | 0.032 | 0.003 | 9.50E-24 |
| Testosterone | rs2270445 | 17 | 8219478 | G | A | 0.487 | 0.017 | 0.003 | 4.60E-10 |
| Testosterone | rs34557412 | 17 | 16852187 | A | G | 0.993 | 0.107 | 0.017 | 1.80E-10 |
| Testosterone | rs1242518 | 17 | 17387899 | T | C | 0.747 | 0.02 | 0.003 | 4.80E-10 |
| Testosterone | rs232159 | 17 | 63708321 | C | T | 0.349 | 0.018 | 0.003 | 1.10E-08 |
| Testosterone | rs34931250 | 17 | 66879927 | C | T | 0.939 | 0.054 | 0.006 | 3.20E-21 |
| Testosterone | rs28421540 | 18 | 3818842 | A | C | 0.714 | 0.031 | 0.003 | 1.90E-24 |
| Testosterone | rs2186945 | 18 | 13921536 | C | T | 0.162 | 0.025 | 0.004 | 2.00E-10 |
| Testosterone | rs34163044 | 18 | 51851616 | A | C | 0.419 | 0.017 | 0.003 | 2.70E-09 |
| Testosterone | rs112367565 | 18 | 71883232 | A | C | 0.046 | 0.049 | 0.007 | 1.30E-13 |
| Testosterone | rs9319895 | 18 | 71896469 | A | G | 0.515 | 0.023 | 0.003 | 2.80E-15 |
| Testosterone | rs117327231 | 18 | 71916636 | A | C | 0.023 | 0.173 | 0.009 | 6.80E-82 |
| Testosterone | rs12977787 | 19 | 1814025 | A | G | 0.541 | 0.015 | 0.003 | 7.20E-09 |
| Testosterone | rs8111359 | 19 | 10471462 | C | T | 0.905 | 0.06 | 0.005 | 1.10E-35 |
| Testosterone | rs4804181 | 19 | 12509536 | C | A | 0.221 | 0.037 | 0.003 | 1.30E-26 |
| Testosterone | rs11673591 | 19 | 41985931 | A | T | 0.252 | 0.028 | 0.003 | 5.70E-19 |
| Testosterone | rs138983180 | 19 | 42914668 | A | G | 0.006 | 0.162 | 0.019 | 2.00E-17 |
| Testosterone | rs7256920 | 19 | 46203083 | G | A | 0.515 | 0.019 | 0.003 | 5.50E-11 |
| Testosterone | rs2879910 | 19 | 48335029 | C | T | 0.481 | 0.016 | 0.003 | 1.70E-08 |
| Testosterone | rs75287599 | 19 | 49517140 | T | C | 0.077 | 0.043 | 0.005 | 1.70E-17 |
| Testosterone | rs11697333 | 20 | 31189078 | T | C | 0.674 | 0.019 | 0.003 | 1.80E-10 |
| Testosterone | rs1883711 | 20 | 39179822 | G | C | 0.968 | 0.05 | 0.008 | 2.00E-09 |
| Testosterone | rs6020423 | 20 | 48909667 | C | T | 0.76 | 0.04 | 0.003 | 1.10E-36 |
| Testosterone | rs6127099 | 20 | 52731402 | A | T | 0.721 | 0.018 | 0.003 | 1.10E-08 |
| Testosterone | rs6100174 | 20 | 57280575 | C | T | 0.625 | 0.02 | 0.003 | 1.70E-13 |
| Testosterone | rs8126001 | 20 | 62711459 | T | C | 0.49 | 0.02 | 0.003 | 6.30E-13 |
| Testosterone | rs2824138 | 21 | 18294462 | C | T | 0.182 | 0.026 | 0.004 | 1.70E-13 |
| Testosterone | rs74652944 | 21 | 33750632 | C | T | 0.008 | 0.183 | 0.016 | 3.70E-31 |
| Testosterone | rs12185851 | 21 | 43372219 | C | T | 0.231 | 0.021 | 0.003 | 3.20E-11 |
| Testosterone | rs9611014 | 22 | 22453513 | C | T | 0.784 | 0.023 | 0.003 | 3.10E-12 |
| Testosterone | rs8184986 | 22 | 29127224 | A | T | 0.866 | 0.023 | 0.004 | 2.40E-08 |
| Testosterone | rs12628709 | 22 | 29647854 | G | A | 0.109 | 0.026 | 0.005 | 1.80E-09 |
| Testosterone | rs4820829 | 22 | 30524248 | C | T | 0.977 | 0.058 | 0.009 | 4.10E-10 |
| Testosterone | rs5751229 | 22 | 42545221 | A | G | 0.228 | 0.025 | 0.003 | 6.10E-14 |
| Testosterone | rs6008259 | 22 | 46633782 | G | A | 0.821 | 0.034 | 0.004 | 2.20E-22 |
| Testosterone | rs7291444 | 22 | 46656246 | T | G | 0.852 | 0.033 | 0.004 | 2.90E-19 |
| Testosterone | rs12837203 | 23 | 70334233 | G | A | 0.676 | 0.019 | 0.003 | 1.80E-11 |
| Testosterone | rs7884765 | 23 | 109883765 | T | C | 0.393 | 0.024 | 0.003 | 2.20E-17 |
| Testosterone | rs67596711 | 23 | 152638744 | T | G | 0.501 | 0.039 | 0.003 | 2.00E-43 |

Abbreviations: SNP, single nucleotide polymorphism; Chr, chromosome; REF, reference allele; ALT, alter allele; EAF, effect allele frequency; SE, standard error.
